# The role of manganese in morphogenesis and pathogenesis of the opportunistic fungal pathogen *Candida albicans*

**DOI:** 10.1371/journal.ppat.1011478

**Published:** 2023-06-26

**Authors:** Asia S. Wildeman, Naisargi K. Patel, Brendan P. Cormack, Valeria C. Culotta

**Affiliations:** 1 The Department of Biochemistry and Molecular Biology, Johns Hopkins University Bloomberg School of Public Health, Baltimore, Maryland, United States of America; 2 Department of Molecular Biology and Genetics, Johns Hopkins University School of Medicine, Baltimore, Maryland, United States of America; University of California Los Angeles David Geffen School of Medicine, UNITED STATES

## Abstract

Metals such as Fe, Cu, Zn, and Mn are essential trace nutrients for all kingdoms of life, including microbial pathogens and their hosts. During infection, the mammalian host attempts to starve invading microbes of these micronutrients through responses collectively known as nutritional immunity. Nutritional immunity for Zn, Fe and Cu has been well documented for fungal infections; however Mn handling at the host-fungal pathogen interface remains largely unexplored. This work establishes the foundation of fungal resistance against Mn associated nutritional immunity through the characterization of NRAMP divalent metal transporters in the opportunistic fungal pathogen, *Candida albicans*. Here, we identify *C*. *albicans* Smf12 and Smf13 as two NRAMP transporters required for cellular Mn accumulation. Single or combined *smf12Δ/Δ* and *smf13Δ/Δ* mutations result in a 10–80 fold reduction in cellular Mn with an additive effect of double mutations and no losses in cellular Cu, Fe or Zn. As a result of low cellular Mn, the mutants exhibit impaired activity of mitochondrial Mn-superoxide dismutase 2 (Sod2) and cytosolic Mn-Sod3 but no defects in cytosolic Cu/Zn-Sod1 activity. Mn is also required for activity of Golgi mannosyltransferases, and *smf12Δ/Δ* and *smf13Δ/Δ* mutants show a dramatic loss in cell surface phosphomannan and in glycosylation of proteins, including an intracellular acid phosphatase and a cell wall Cu-only Sod5 that is key for oxidative stress resistance. Importantly, *smf12Δ/Δ* and *smf13Δ/Δ* mutants are defective in formation of hyphal filaments, a deficiency rescuable by supplemental Mn. In a disseminated mouse model for *candidiasis* where kidney is the primary target tissue, we find a marked loss in total kidney Mn during fungal invasion, implying host restriction of Mn. In this model, *smf12Δ/Δ* and *smf13Δ/Δ C*. *albicans* mutants displayed a significant loss in virulence. These studies establish a role for Mn in *Candida* pathogenesis.

## Author summary

Nutritional immunity is the host withholding of nutrients including metals from microbial pathogens during infection. A great deal is understood regarding nutritional immunity for Zn, Cu and Fe during fungal infections, but very little is known about Mn and fungal pathogenesis. Using the opportunistic and polymorphic fungal pathogen *Candida albicans* as a model, we describe a host immune response involving Mn during *Candida* infection and demonstrate the importance of Mn for fungal virulence. Our studies reveal two divalent metal transporters in *C*. *albicans*, namely Smf12 and Smf13, that are needed for Mn accumulation, for Mn dependent enzymes involved in oxidative stress resistance and protein glycosylation, for proper formation of the mannose layer of the cell wall and for hyphal morphogenesis. Using a mouse model of systemic *candidiasis*, we document a restriction in host Mn that occurs through decreased Mn levels upon *Candida* invasion of the kidney. *C*. *albicans* survives this host restriction of Mn and both Smf12 and Smf13 contribute to virulence. These findings on Mn at the host-fungal pathogen interface are likely to be extrapolated to other fungal pathogens of public health importance.

## Introduction

All living organisms acquire metals such as Fe, Cu, Zn and Mn as essential trace nutrients, as these metals are employed as co-factors for nearly half of all enzymes [1]. The mammalian immune system exploits this nutritional requirement by withholding metals at sites of infection to starve invading microbes through a response known as nutritional immunity [2]. Successful pathogens can counteract host nutritional immunity using mechanisms to maintain metal nutrient levels in a restrictive environment. How bacterial and fungal pathogens acquire Fe, Cu, and Zn in the face of host-imposed metal sequestration has been widely studied [2–6].

Much of what is currently understood regarding microbial Mn during infection comes from studies with bacterial pathogens. Pathogenic bacteria require Mn to guard against the oxidative attack of host immunity, primarily through Mn-superoxide dismutase (SOD) enzymes, and Mn is also needed for bacterial carbon and nucleic acid metabolism [7–9]. During infection, the host can restrict Mn for the bacterial pathogen through neutrophil release of metal binding calprotectin [10–15] or through macrophages that employ the divalent metal transporter NRAMP1 to create a Mn-starvation environment for a phagocytosed microbe [16]. NRAMP1 is the prototype of a large family of proton-coupled divalent metal transporters that are well-conserved from bacteria to eukaryotes [17]. In fact, certain bacterial versions of NRAMP (so-called MntH), have been shown to counteract host withholding of Mn and promote microbial growth and pathogenesis in the Mn-limited immune environment [7, 8, 18–20].

In contrast, very little is known about Mn and virulence for fungal pathogens, which are responsible for an estimated 2 million life threatening infections per year worldwide [21]. As with bacteria, eukaryotes including fungi rely on Mn as co-factor for SOD enzymes and multiple transferases, hydrolases and lyases have been shown to utilize Mn. Either Mn or Mg can serve as co-factors for glutamine synthetase, pyruvate carboxylase and arginase [22, 23], and Mn is the physiological activator of TOR in yeast and mammals [24]. Additionally, all eukaryotes require Mn as a co-factor for numerous glycosyl transferases in the Golgi for protein processing in the secretory pathway. Mn is delivered to the Golgi by a Ca and Mn P-type ATPase known in fungi as Pmr1, and mutations in *PMR1* or its partner *GDT1* have been associated with defects in morphogenesis and differentiation and in virulence of certain fungal pathogens [25–27]. However, since Pmr1 transports both Ca and Mn, the specific contribution of Mn to these defects is not well understood.

By genomic analyses, NRAMP transporters occur widely throughout pathogenic fungi with the potential to transport a range of divalent metals [28]. Regarding fungal requirement for Mn, much of what is currently known about cell surface Mn transporters stems from studies in the non-pathogenic bakers’ yeast *Saccharomyces cerevisiae*. Two *S*. *cerevisiae* NRAMP transporters known as Smf1 and Smf2 provide the cell with Mn needed for antioxidant protection and protein glycosylation [29–32]. In the human fungal pathogen *Cryptococcus neoformans*, NRAMP mutations have been linked to Cd resistance, and in the *Aspergillus orzyae* plant pathogen, loss of a NRAMP transporter causes defects in differentiation through mechanisms possibly involving Zn, Fe and/or Mn [33, 34].

The opportunistic fungal pathogen *C*. *albicans* represents an ideal system to study metals at the host-fungal pathogen interface. *C*. *albicans* causes superficial mucosal infections as well as invasive infections that carry a mortality rate of approximately 30% despite antifungal availability [21]. *C*. *albicans* is polymorphic, and morphological plasticity ranges from a rounded yeast-form to extended hyphal filaments that enable host cell penetration and tissue invasion. *C*. *albicans’* success as a pathogen in part stems from its ability to adapt to diverse and changing host environments with sophisticated mechanisms for acquiring nutrients and micronutrients including metal ions in the face of host sequestration. The adaptation of *C*. *albicans* to host withholding of Zn, Fe and Cu during infection [5, 6, 35–43] has been described, but no studies have examined Mn in this regard. *C*. *albicans* is known to require Mn for both cytosolic and mitochondrial Mn-Sods and for N-linked glycosylation by Mn-mannosyltransferases (MNT) [27, 40, 44, 45]. The extensive protein mannosylation by *Candida* MNTs forms the outer mannan layer of the cell wall that is integral for cell structure and immune recognition [46]. How *C*. *albicans* accumulates Mn for cell growth and pathogenesis is unknown.

Here we describe for the first time a Mn transport system in *C*. *albicans* involving two NRAMP Mn transporters, Smf12 and Smf13. We find both proteins are needed for full activity of Mn-Sod enzymes, for maturation of proteins such as Cu-only Sods through mannosylation, for assembly of the mannose cell wall layer and, for hyphal morphogenesis. Moreover, we document a host response to fungal infection that involves withholding of tissue Mn at the site of kidney invasion, and demonstrate an essential role for fungal Mn during pathogenesis in a disseminated mouse model for *candidiasis*. Our studies establish an important role for Mn at the host-fungal pathogen interface.

## Results

### A role for C. albicans Smf12 and Smf13 in Mn accumulation

**Fig 1:**
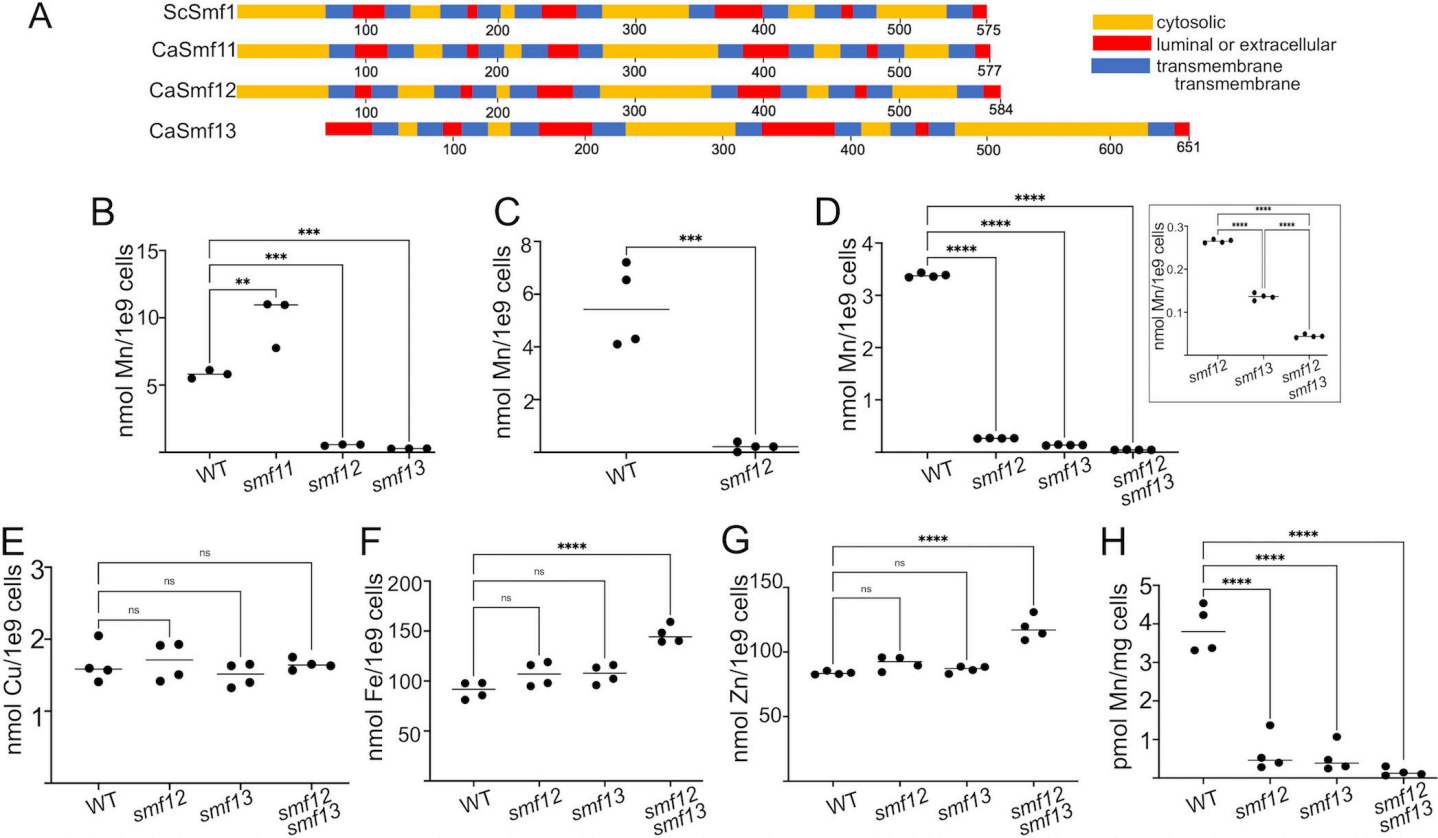
*C*. *albicans* SMF11, SMF12 and SMF13 as candidate metal transporters. (A) Predicted topology of *C*. *albicans* Smf11, Smf12 and Smf13 in comparison to *S*. *cerevisiae* Smf1 as determined by TOPCONS software. The numbers designate amino acid position. (B-H) Intracellular levels of Mn (B-D and H) or Cu (E) or Fe (F) or Zn (G) were measured in the indicated strains grown as either yeast-form cells in YPD (B-G) or in hyphal cells following 3 hrs growth in IMDM (H). Inset in D shows results with *smf* mutants alone and expanded Y axis scale. Strains utilized: (B, D-H) WT, SC5314; *smf11* (AW001), *smf12Δ/Δ* (AW002), *smf13Δ/Δ* (AW003), *smf12Δ/Δ smf13Δ/Δ* (AW004); (C) WT, DAY286; *smf12*, *smf12Δ/Δ* derivative of DAY286. Significance was determined using one-way ANOVA with a Tukey posttest, or in the case of the inset, *t* test. ****p<0.0001, ***p<0.001, **p<0.01, *p<0.05. ns p>0.05. Results are shown for 3–4 biological replicates over 2 experimental trials.

To define the impact of Mn limitation in *C*. *albicans* cell function and virulence we sought to identify *C*. *albicans* Mn transporters. The distantly related Ascomycete *S*. *cerevisiae* relies on the divalent-metal NRAMP transporters Smf1 and Smf2 for high affinity transport of Mn from the environment [29–32]. *C*. *albicans* has 4 paralogues of Smf1/2, namely Smf3 (orf19.2069), Smf11 (orf19.4690), Smf12 (orf19.2270) and a fourth orf19.5022 that we have named Smf13. Smf3 has previously been documented as a vacuolar transporter for Fe [47], while to our knowledge, Smf11, Smf12 and Smf13 have not been previously characterized. *C*. *albicans* Smf11, Smf12 and Smf13 share ≈ 50% amino acid identity with *S*. *cerevisiae* Smf1 (S1 Fig). The predicted topology of *C*. *albicans* Smf11 and Smf12 are very similar to *S*. *cerevisiae* Smf1 (Fig 1A), while *C*. *albicans* Smf13 is missing N-terminal sequences including the first transmembrane domain and contains an expanded cytosolic loop near the C-terminus (Fig 1A).

To test whether *SMF11*, *SMF12* and *SMF13* function in Mn accumulation, we used CRISPR-Cas9 [48] to generate homozygous null mutations for each gene in the SC5314 background. The resultant strains were all viable and we analyzed the intracellular Mn concentration in each. In yeast-form cells grown in enriched YPD media, we find that *smf12Δ/Δ* and *smf13Δ/Δ* mutants exhibit ≈10 and 20–25 fold decreases respectively in intracellular Mn in comparison to the wildtype strain, while *smf11Δ/Δ* mutants if anything showed an elevation in total Mn (Fig 1B). The reduction in total cellular Mn with *smf12Δ/Δ* mutations was likewise seen in an independent strain background, DAY286 (Fig 1C). We additionally created a double *smf12Δ/Δ smf13Δ/Δ* mutant and assessed growth and intracellular Mn levels in comparison to the single mutants. The strain is viable although exhibits a somewhat slowed growth (S2 Fig). As seen in Fig 1D and inset, the effect of combining *smf12Δ/Δ* and *smf13Δ/Δ* mutations was additive and total intracellular Mn levels decreased approximately 75-fold in the *smf12Δ/Δ smf13Δ/Δ* double mutant compared to wildtype cells under the same growth conditions. Importantly, the effects of the *smf12Δ/Δ* and *smf13Δ/Δ* mutations seem specific for Mn, as intracellular levels of Cu, Fe and Zn were not diminished in these strains (Fig 1E–1G). If anything, Fe and Zn levels tended to increase with decreasing Mn levels (Fig 1F and 1G). Our YPD media contains ≈1.0 μM Mn, consistent with published studies [49]. When YPD is supplemented with 10–100 fold higher levels of Mn, the *smf12Δ/Δ* and *smf13Δ/Δ* strains accumulate Mn near WT levels, with the *smf13Δ/Δ* mutant requiring higher levels of supplementation than *smf12Δ/Δ* strains (S3 Fig).

*C*. *albicans* is polymorphic, and we examined whether the Mn accumulation defects we observed in yeast-form cells also occurred in hyphal cells. For these studies, cells were induced to undergo morphogenesis by growth in IMDM (Iscove’s Modified Dulbecco’s Medium) in which high levels of amino acids in the media trigger hyphal formation [50, 51]. IMDM contains ≈5 nM Mn and as was observed with yeast-form cells, total cellular Mn was greatly reduced in hyphal cells for both the *smf12Δ/Δ* and *smf13Δ/Δ* single and double mutants (Fig 1H).

### Loss of Mn-dependent enzymes in C. albicans smf12Δ/Δ and smf13Δ/Δ mutants

**Fig 2:**
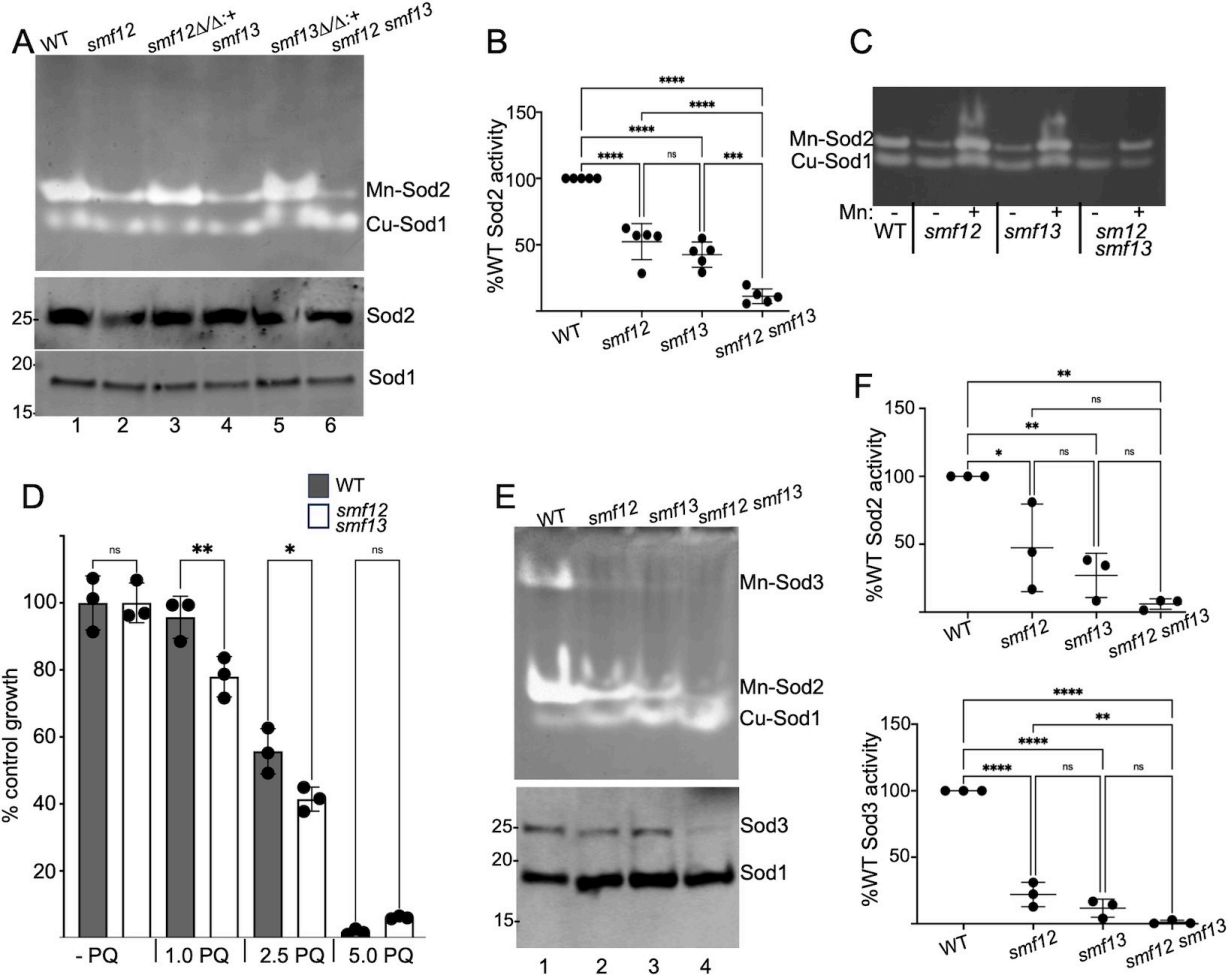
Mn containing Sod enzymes are defective in *smf12Δ/Δ* and *smf13Δ/Δ* mutants. Yeast-form cells were obtained by growth in YPD (A-D) or cells were induced to form hyphae in IMDM for 3 hours (E,F). Where indicated (part C, Mn: +), cultures were supplemented with 50 μM MnCl_2_. (A-C and E,F) Lysates were prepared and were subjected to non-denaturing gel electrophoresis and NBT staining for Sod activity (A,C and E top) or to denaturing gel electrophoresis and immunoblot analysis of indicated Sod proteins (A and E bottom) as described in *Materials and Methods*. Molecular weight markers of immunoblots are indicated on left margins. Results from 3–5 independent experimental trials were quantified for Sod2 activity in YPD grown cells and IMDM hyphae (B, F top respectively) and for Sod3 activity in IMDM hyphal cells (F bottom). (D) Three independent cultures of the indicated strains were grown in the presence of the designed concentrations of paraquat as described in *Materials and Methods*, and total growth following 20 hrs (14–18 cell doublings) was determined by OD_600_. Strains utilized are as described in Fig 1D–1H; *smf12Δ/Δ*:*+* (AW005) and *smf13Δ/Δ*:*+* (AW006) are *smf12Δ/Δ* and *smf13Δ/Δ* nulls complemented with a single copy of the *SMF12* and *SMF13* gene.

Two superoxide dismutase (SOD) enzymes in *C*. *albicans* require Mn: a mitochondrial Mn-Sod2 that is constitutively expressed, and a cytosolic Mn-Sod3 that is specifically induced with low Cu conditions to substitute for loss of Cu/Zn-Sod1 [40]. In yeast-form cells grown in Cu replete conditions we observe a decrease in activity of mitochondrial Mn-Sod2 in both *smf12Δ/Δ* and *smf13Δ/Δ* single mutants (Fig 2A top, lanes 2 and 4) as well as in the *smf12Δ/Δ smf13Δ/Δ* double mutant (lane 6). In contrast to inhibition of Mn-Sod2 activity, there is no loss in activity of cytosolic Cu/Zn Sod1 (Fig 2A, top). Quantification of various experimental trials revealed decreases in Mn-dependent Sod2 activity of ≈40% and 50% for the single mutants and ≈90% for the double *smf12Δ/Δ smf13Δ/Δ* mutant (Fig 2B). The loss in Sod2 activity occurs with relatively constant levels of Sod2 protein (Figs 2A, middle and S4A), and can be rescued by supplementing the growth media with excess Mn (50 μM) (Fig 2C). Thus, the low Sod2 activity of *smf12Δ/Δ* and *smf13Δ/Δ* mutants results from a deficiency in the Mn co-factor for this enzyme. *C*. *albicans sod2Δ/Δ* mutants are highly sensitive to the superoxide generating agent paraquat [44], and we tested whether the reduced Sod2 activity of the *smf* mutants leads to a similar paraquat hypersensitivity. As seen in Fig 2D, the overall dose response to paraquat was very comparable with WT and *smf12Δ/Δ smf13Δ/Δ* strains, and total growth at 1.0 and 2.5 μM paraquat over >14 cell divisions varied no more than 20% in the two strains. This relative resistance to paraquat seen in the *smf12Δ/Δ smf13Δ/Δ* mutant (which retains ≈10% Sod2 enzyme activity) is consistent with previously published studies that very little SOD activity is needed to guard against superoxide toxicity [52].

We also examined SOD activity in hyphal cells grown in IMDM. Mn-dependent Sod2 activity likewise decreases in hyphal cells of the *smf12Δ/Δ* and *smf13Δ/Δ* mutants, with an additive effect in the *smf12Δ/Δ smf13Δ/Δ* double mutant (Fig 2E top and 2F). The cytosolic Mn-Sod3 is induced only under conditions of Cu limitation [40]. Since IMDM is commercially prepared without heavy metal supplementation [53], Cu is sufficiently low in IMDM to induce *C*. *albicans* expression of cytosolic Mn-Sod3 (Fig 2E top, lane 1). Consistent with our findings for Sod2, activity of the cytosolic Mn-Sod3 also decreases in the *smf12Δ/Δ* and *smf13Δ/Δ* mutants, with a near complete loss in the *smf12Δ/Δ smf13Δ/Δ* double mutant (Fig 2E top and 2F bottom). The double *smf12Δ/Δ smf13Δ/Δ* mutant also consistently shows a loss in Sod3 protein and thus the decrease in Sod3 activity in this strain cannot be totally ascribed to loss of the Mn co-factor (Figs 2E bottom and S4B). Curiously in IMDM grown cells, we often observe an increase in cytosolic Cu/Zn Sod1 activity in the *smf12Δ/Δ* and *smf13Δ/Δ* mutants (Figs 2E top and S4C). Although the mechanism is unknown, cells may enhance activity of cytosolic Cu-Sod1 to compensate for the loss in cytosolic Mn-Sod3. Collectively these findings are consistent with a role for Smf12 and Smf13 in Mn activation of SOD enzymes.

**Fig 3:**
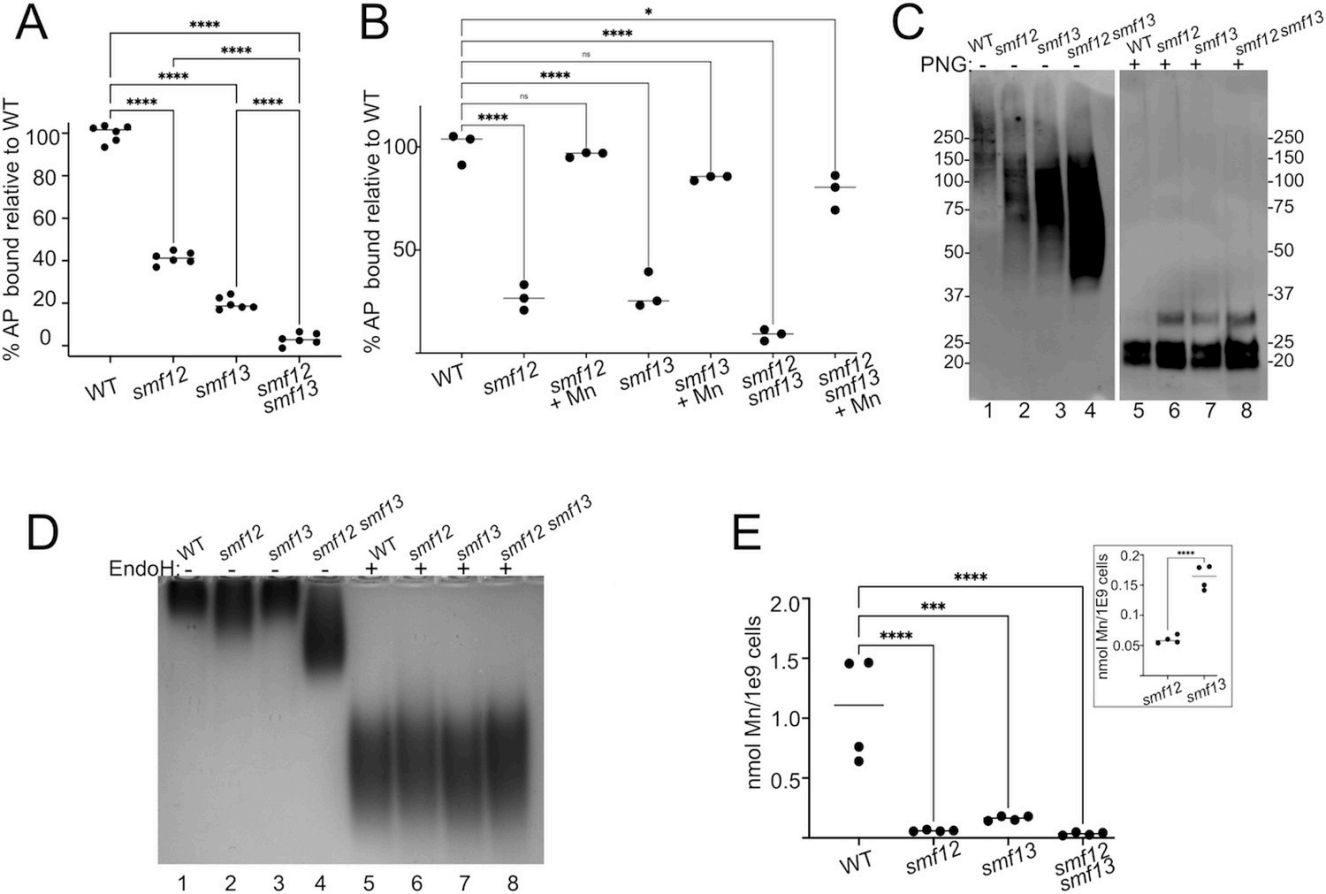
Wide spread defects in protein mannosylation in *smf12Δ/Δ* and *smf13Δ/Δ* mutants. (A,B) Total cell surface phosphomannas were quantified in the designated strains by Alcian Blue (AB) binding and plotted as a percentage of WT AP binding as described in *Materials and Methods*. Where indicated, cultures were supplemented with 50 μM MnCl_2_. Data shows results from three—six biological replicates over two experimental trials. (C) Immunoblot analysis of Sod5 from the cell wall was carried out in the indicated strains as described in *Materials and Methods*. Samples were either treated (PNG: +) or not treated (PNG: -) with the endoglycosidase PNGase F prior to electrophoresis. Enhanced mobility of PNG:- samples reflects defects in Sod5 mannosylation. (D,E) The indicated strains were grown in a phosphate deficient media and analyzed for (D) intracellular acid phosphatase activity by a native gel assay as described in *Materials and Methods* or for (E) cellular Mn levels. (D) Where indicated (Endo H: +), samples were treated with endoglycosidase Endo H prior to electrophoresis. Enhanced mobility of acid phosphatase in part indicates defective protein mannosylation. Strains are as described in Fig 1D–1H and statistical significance determined as in Fig 1.

Manganese also needs to be distributed to the Golgi for activation of numerous mannosyltransferases (MNT) that glycosylate proteins in the secretory pathway [45]. Protein mannose chains have a terminal phosphate that readily reacts with Alcian Blue, a reporter of cell surface mannosylation [45, 54, 55]. As seen in Fig 3A, *smf12Δ/Δ* and *smf13Δ/Δ* mutants show a dramatic decrease in cell surface phosphomannan compared to wildtype cells, and the effect is additive in the *smf12Δ/Δ smf13Δ/Δ* double mutant. This defect is due to Mn depletion since the poor cell surface mannosylation was rescued by growth in elevated Mn (Fig 3B). Because Alcian Blue binding is an indirect reporter of O-linked and N-linked protein mannosylation [56], we directly assessed protein mannosylation by surveying the glycosylation state of individual proteins.

The Cu-only superoxide dismutase Sod5 is a mannosylated GPI-anchor protein that is abundantly expressed in the cell wall of hyphal cells [53]. Sod5 plays an important role in protecting *C*. *albicans* against the oxidative burst of host phagocytes and in fungal virulence [57–61]. On immunoblots, native Sod5 from hyphal cells grown in IMDM appears as a >150 kDa heavily glycosylated protein (Fig 3C lane 1) that will collapse into ≈20kDa species when treated with the deglycosylase PNGase F (Fig 3C, lane 5). The Sod5 signal also gains intensity on immunoblots following deglycosylation due to improved antibody recognition [62]. We observe that cell wall Sod5 from *smf12Δ/Δ* and *smf13Δ/Δ* mutants migrates faster on immunoblots without deglycosylation by PNGase, and intensity of the Sod5 signal increases, especially in the *smf12Δ/Δ smf13Δ/Δ* double mutant (Fig 3C lanes 2–4). By comparison, immunodetection and electrophoretic mobility of Sod5 that was deglycosylated by PNGase F is the same in WT and *smf* mutant strains (Fig 3C lanes 5–8), the only difference being an increase in a ≈30 kDa species that has previously been shown to be Sod5 with a remnant beta glucan [53]. We are unsure of the basis for the increase in this band, but it may be an indirect effect of altered cell wall structure in the *smf* mutants. In any case, the effects of *smf* mutations on Sod5 in the absence of PNGase (Fig 3C lanes 1–4) are indicative of defective mannosylation and demonstrate an important role for Mn in maturation of Cu-only SODs.

We also examined glycosylation of an intracellular mannosylated protein. Acid phosphatase (AP) is a heavily N-linked mannosylated protein that in yeast species, can reside in the Golgi, secretory vesicles and vacuole [63]. In the experiment of Fig 3D, cells were grown in media lacking phosphate to induce AP expression, and AP glycosylation was examined by electrophoretic mobility as previously described [27]. We observe that mobility of enzymatically active AP increases with the *smf12Δ/Δ* mutant and the defect is more severe in the *smf12Δ/Δ smf13Δ/Δ* double mutant (Fig 3D, lanes 2 and 4). This change in electrophoretic mobility is due to changes in glycosylation since mobility of deglycosylated AP samples (treated with Endo H) is the same in WT and *smf* mutant strains (lanes 5–8). Unexpectedly, there was minimal aberration in AP electrophoresis without Endo H treatment in the case of the *smf13Δ/Δ* single mutant (Fig 3D, lane 3). Nevertheless, the increased AP mobility with the *smf12Δ/Δ smf13Δ/Δ* double compared to the single *smf12Δ/Δ* mutant (Fig 3D lanes 2 and 4) indicates that both Smf12 and Smf13 contribute to AP glycosylation, with Smf12 playing a more prominent role under these conditions. The extent of the glycosylation defect correlates nicely with results of cellular Mn levels under these low phosphate growth conditions: *smf12Δ/Δ* mutants accumulate lower Mn than *smf13Δ/Δ* cells (Fig 3E and inset). The defects observed with AP mannosylation corroborate findings with Sod5 mannosylation and cell surface phosphomannan, all indicating that Golgi Mn-mannosyltransferases are broadly defective in *smf12Δ/Δ* and *smf13Δ/Δ* mutants under three distinct growth conditions.

### Hyphal morphogenesis defect of smf12Δ/Δ and smf13Δ/Δ mutants

**Fig 4:**
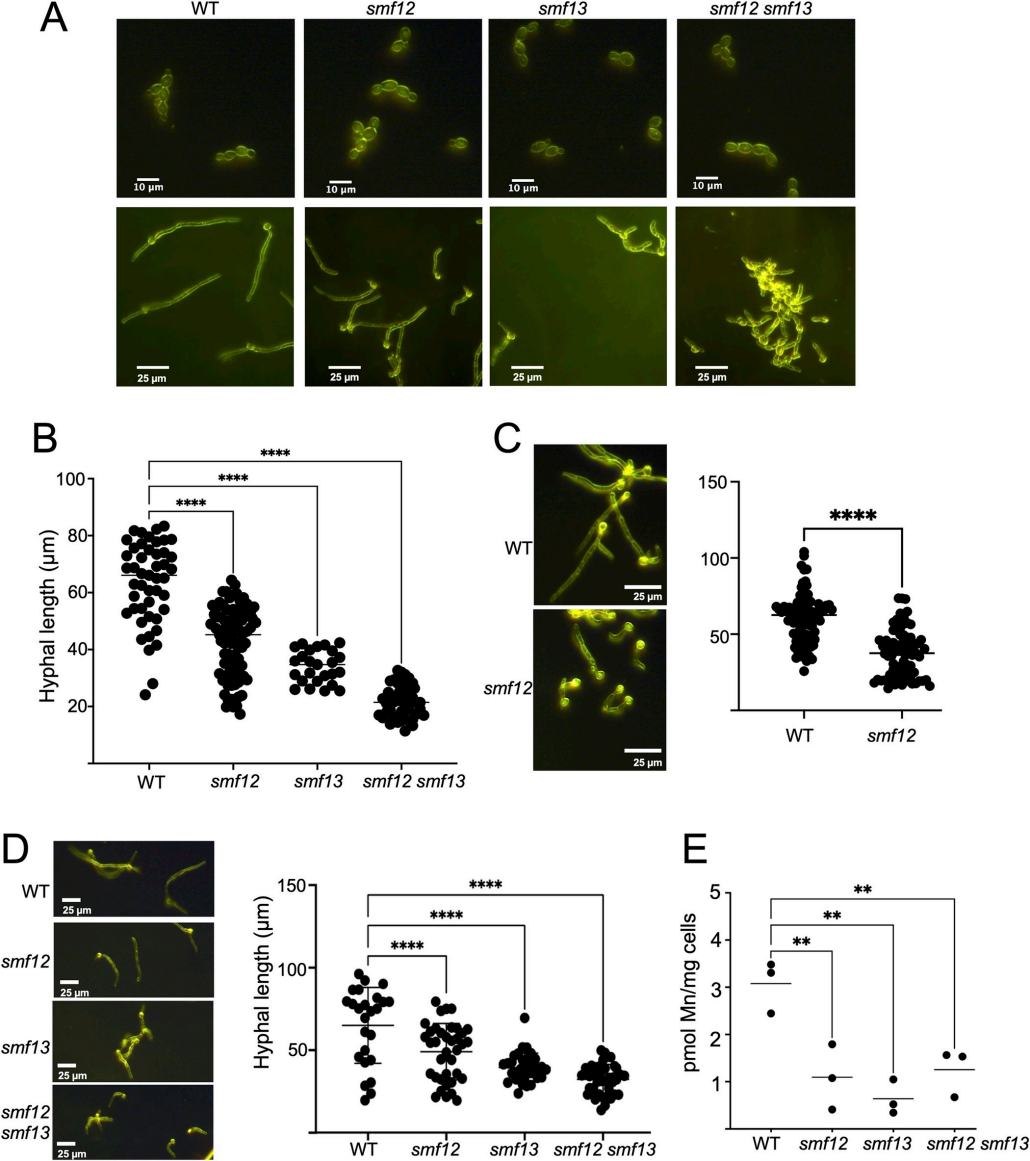
Hyphal defects in *smf12Δ/Δ* and *smf13Δ/Δ* mutants. The indicated strains from either the SC5314 (A,B,D,E) or DAY286 (C) background were imaged by dark field microscopy as either yeast-form cells (A top) or as hyphal cells (A bottom and C and D left) following 3 hours growth in IMDM (A-C) or in serum (D). Hyphal length was quantified using ImageJ software (B and C and D right). (E) Serum hyphal cells from three independent trials were analyzed for Mn accumulation by AAS. Strains are as described in Fig 1 and significance determined by one-way ANOVA with a Tukey posttest (B, D right, E), or *t* test (C). ****p<0.0001; **p≤0.01.

We noticed that *smf12Δ/Δ* and *smf13Δ/Δ* mutants exhibited an aberrant morphology. When growing as yeast-form cells, we did not find any differences in morphology between wildtype and the *smf12Δ/Δ* and *smf13Δ/Δ* mutants (Fig 4A top). By contrast in media that promotes hyphal growth, both *smf12Δ/Δ* and *smf13Δ/Δ* mutants exhibit a change in the length and overall morphology of hyphae. In the experiment of Fig 4A bottom and 4B, cells were induced to undergo morphogenesis for 3 hours in IMDM, conditions where wildtype cells generate extended hyphae. By comparison, the *smf12Δ/Δ* and *smf13Δ/Δ* mutants have overall shorter hyphal lengths, with a more pronounced defect in *smf13Δ/Δ* mutants, and an additive effect of the double *smf12Δ/Δ smf13Δ/Δ* mutation on shortening hyphae (Fig 4A and 4B). Consistent with this result in the SC5314 background, DAY286 derived *smf12Δ/Δ* strains also exhibited shorter hyphae (Fig 4C). The experiments of Fig 4A–4C were conducted in IMDM media containing ≈5 nM Mn and we additionally tested whether this defect holds true in serum, a more physiological inducer of hyphal morphogenesis. Fetal calf serum contains ≈350–400 nM Mn, and all three *smf* mutants exhibited a defect in morphogenesis induced by serum and lowered Mn accumulation (Fig 4D and 4E). As with IMDM, the morphogenic defect in serum was more pronounced with *smf13Δ/Δ* compared to *smf12Δ/Δ* cells, and the double mutant *smf12Δ/Δ smf13Δ/Δ* was most severely impaired (Fig 4D).

**Fig 5:**
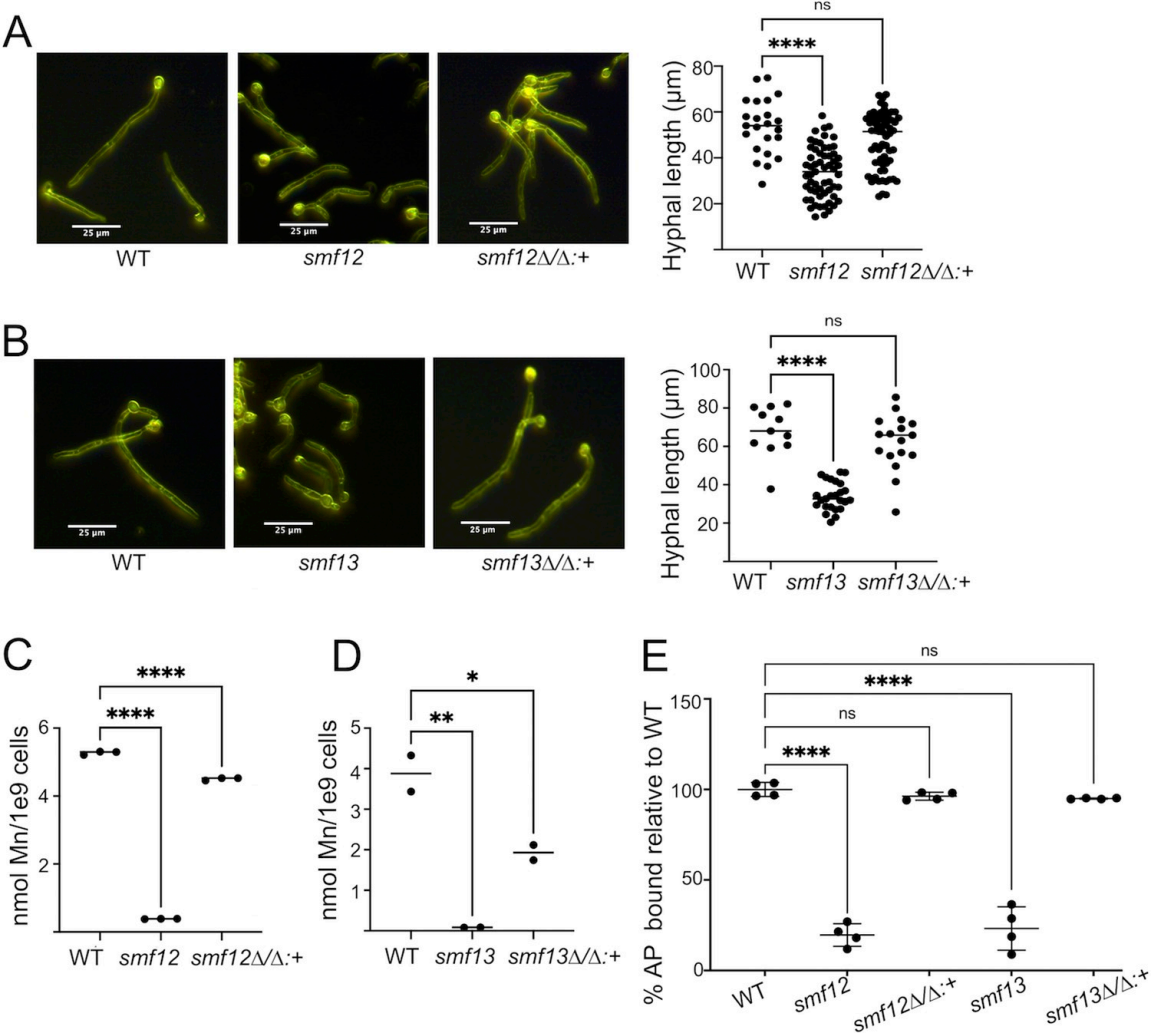
Complementation of *smf12Δ/Δ and smf13Δ/Δ* mutations by single copy of *SMF12 or SMF13*. The indicated strains were induced to form hyphae by growth in IMDM for 3 hours. Cells were analyzed for (A,B) hyphal length as in Fig 4, for (C,D) intracellular Mn, and for (E) cell surface phosphomannans by Alcian Blue binding as in Fig 3A. Graphed results are from two-four independent cultures and significance determined by one-way ANOVA with a Tukey posttest. ****p<0.0001, ***p<0.001, **p<0.01, *p<0.05. ns p>0.05. Strains are as described in Fig 1D–1H; *smf12Δ/Δ*:*+* (AW005) and *smf13Δ/Δ*:*+* (AW006) are *smf12Δ/Δ* and *smf13Δ/Δ* nulls complemented with a single copy of *SMF12* and *SMF13*, respectively.

The morphogenic defects of the *smf12Δ/Δ* and *smf13Δ/Δ* mutants were reversed upon introducing a single copy of *SMF12* or *SMF13* into the corresponding homozygous null strains (Fig 5A and 5B). Interestingly, this single insertion of *SMF12* or *SMF13* only partly restored Mn levels to that of wildtype (Fig 5C and 5D), yet this partial restoration was sufficient to totally reverse defects in Mn-Sod (Fig 2A), cell surface mannosylation (Fig 5E), as well as hyphal deficiencies (Fig 5A and 5B) of the *smf12Δ/Δ* and *smf13Δ/Δ* mutants. Presumably only a fraction of Smf12 and Smf13 derived Mn is required for these cellular activities.

**Fig 6:**
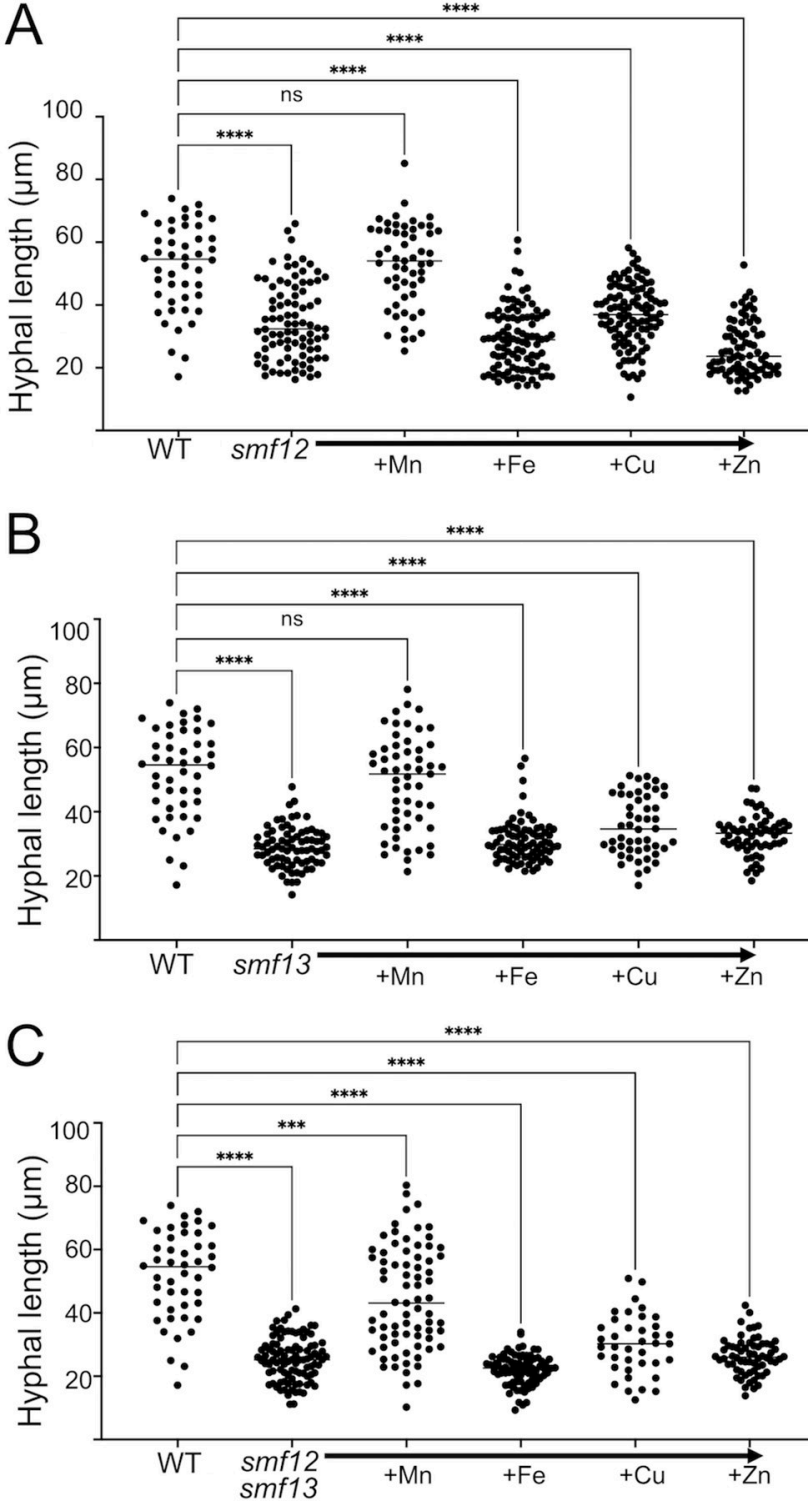
Rescue of the hyphal morphology defect by supplementation of Mn salts but not other metals. The indicated strains were induced to form hyphae for 3 hours in IMDM supplemented with 10 μM levels of the chloride salts for Mn, Fe, Cu and Zn. Hyphal morphology was imaged and quantified as in Fig 4. Significance determined by one-way ANOVA with a Tukey posttest. ****p<0.0001, ns p>0.05.

To confirm that the hyphal defect of *smf12Δ/Δ* and *smf13Δ/Δ* mutants is due to Mn deficiency and not other metals, we tested whether we could restore the morphological defect by supplementing the growth media with Mn, Cu, Fe and Zn. As seen in Fig 6, the hyphal growth deficiency of the *smf12Δ/Δ* and *smf13Δ/Δ* single mutants was fully rescued with 10 μM Mn supplementation, but not by supplementation with the same levels of Fe, Cu and Zn. Similar to our findings here, Mn supplements have also been reported to rescue a morphological deficiency associated with mutant NRAMP in *Aspergillus niger* [34, 64].

**Fig 7:**
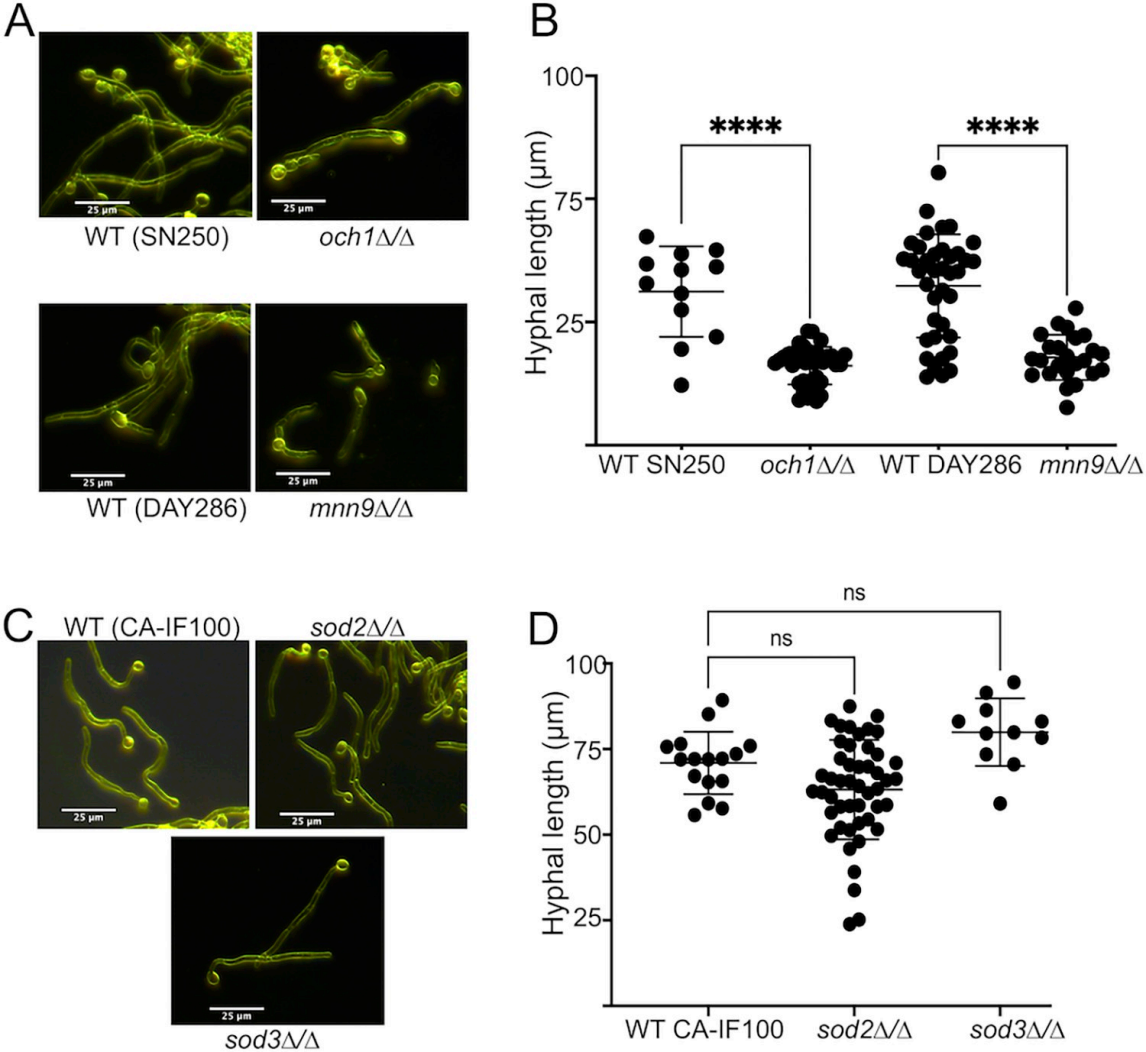
Mutants defective in protein mannosylation, but not Mn Sod activity, exhibit a hyphal defect similar to that of *smf12Δ/Δ* and *smf13Δ/Δ* strains. The indicated strains were induced to form hyphae by 3 hours growth in IMDM and imaged by dark field microscopy and hyphal length quantified as describes in Fig 4.

*C*. *albicans* requires Mn for numerous biochemical processes, and it is challenging to identify the particular Mn metalloenzyme(s) that account for the defect in hyphal morphogenesis described here. With that caveat, we note that other mutants affecting mannosylation also present with defects in hyphal morphogenesis. *OCH1* and *MNN9* encode the first and second steps in outer chain branching of N-linked glycans of mannose chains [65, 66] and *och1Δ/Δ* and *mnn9Δ/Δ* mutants have previously been shown to have hyphal defects in serum [66, 67]. We find these mutants also exhibit a hyphal defect in IMDM similar to our *smf12Δ/Δ* and *smf13Δ/Δ* mutants (Fig 7A and 7B). In contrast, *sod2Δ/Δ* and *sod3Δ/Δ* mutants have no detectable hyphal growth defects under these conditions (Fig 7C and 7D). Based on this observation, it is possible that the loss in MNT activity in *smf12Δ/Δ* and *smf13Δ/Δ* mutants results in the hyphal growth defect we observe. We cannot exclude other possibilities, however, since numerous enzymes use Mn as co-factor. For example, recent studies in *S*. *cerevisiae* have shown that TORC1 is activated by Mn [24]. Since TOR activity is essential for *C*. *albicans* morphogenesis [68], Mn may also contribute to hyphal development through mechanisms involving TOR.

### Host and fungal mn during a mouse model of systemic candidasis

**Fig 8:**
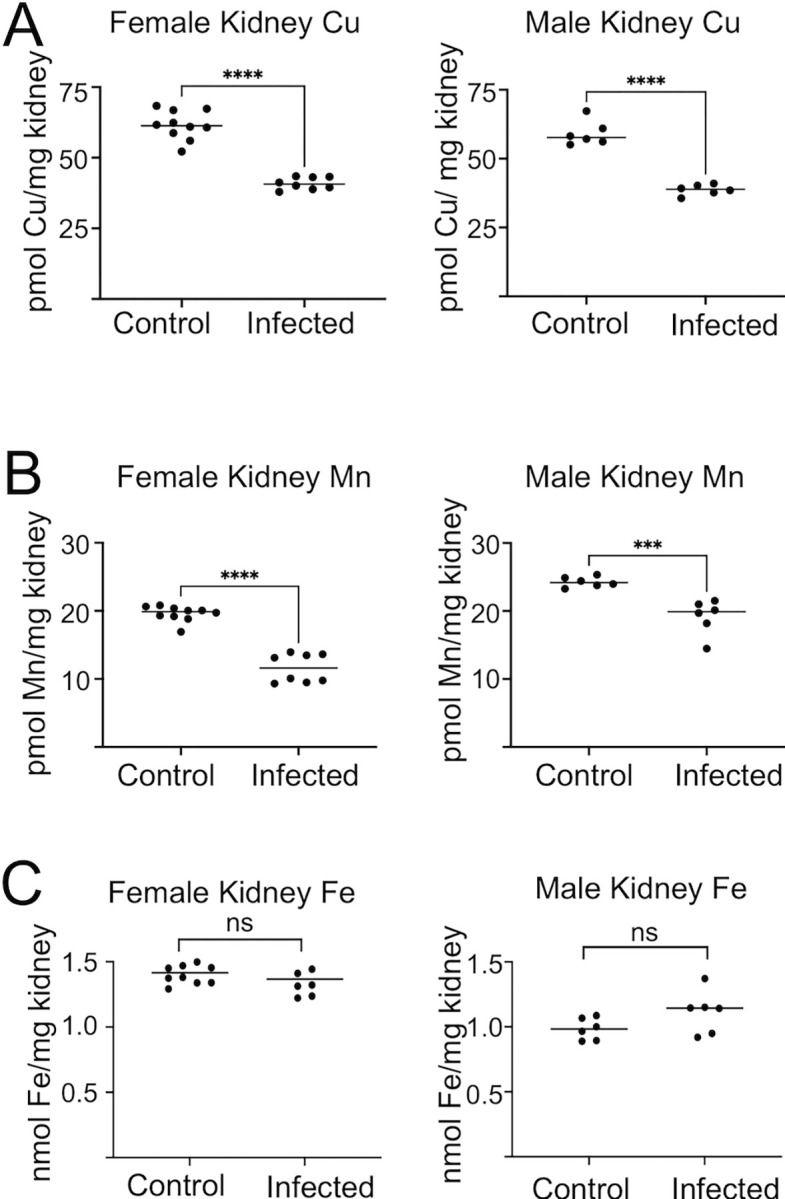
Changes in kidney Mn and Cu during fungal invasion. 11-week old female or male BALB/c mice were infected with 5x10^5^ SC5314 *C*. *albicans* cells. Kidneys were harvested from 72 hour infected mice and uninfected controls and subjected to total Cu (A) and Mn (B) and Fe (C) analysis as described in *Materials and Methods*. Results are from four or five mice per group. Significance was determined by *t-*test. ****p<0.0001, ***p<0.001.

To test the importance of fungal Mn during pathogenesis, we used a disseminated mouse model of *candidiasis* where kidney is the primary target of *C*. *albicans* infection. During kidney invasion, there are numerous changes in host metals indicative of nutritional immunity including withholding of Cu, Fe and Zn micronutrients from the fungal pathogen [4–6, 35, 37, 40]. Mn dynamics in this model has not been previously explored and how tissue Mn is affected during any condition of infection and inflammation is poorly understood [69]. We surveyed Fe, Cu and Mn levels in the kidneys of male and female BALB/c mice that were uninfected and infected 72 hours with *C*. *albicans*. Consistent with previous studies [5, 36, 37, 40], both males and females exhibited a decrease in kidney Cu at 72 hours post-infection (Fig 8A). Remarkably, we observed the same is true for kidney Mn, where the infected kidney loses up to 40% of its total Mn at 72 hours (Fig 8B). Both males and females exhibit this Mn loss, the only difference being somewhat lower levels of Mn in the uninfected control kidneys of females (Fig 8B). Not all kidney metals exhibit this decline during infection and as seen in Fig 8C, Fe levels remain constant. To our knowledge, the loss in kidney Mn with infection is the first evidence of the host reducing whole tissue Mn in response to fungal invasion.

**Fig 9:**
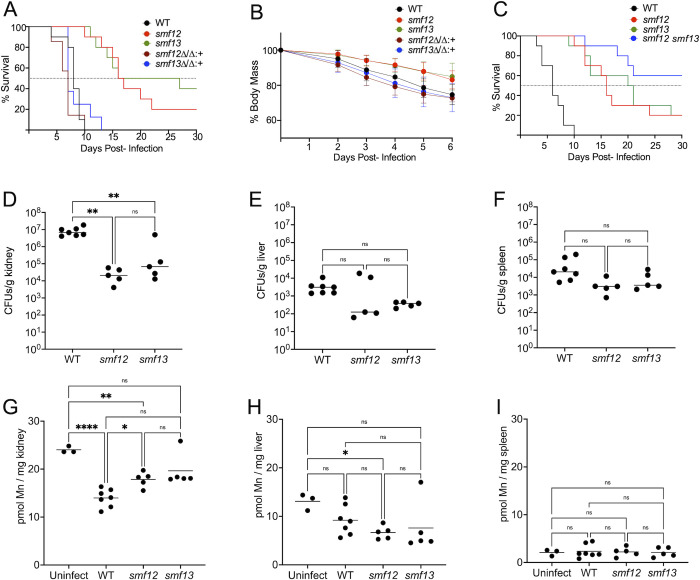
*SMF12* and *SMF13* are important for virulence in a disseminated mouse model of *candidiasis*. (A-C) 11-week (A,B) or 14-week (C) old BALB/c male mice were infected with 2x10^5^ cells of the indicated strains of *C*. *albicans*. (A,C) Survival of 7–10 mice per group was monitored for up to 30 days post infection; dotted line represents median survival. By a log-rank (Mantel-Cox) test, the difference in survival between WT and *smf12Δ/Δ* and *smf13Δ/Δ* mutant infected mice was statistically significant in both A and C (p <0.0001), as is the difference between *smf12Δ/Δ* and *smf12Δ/Δ smf13Δ/Δ* infected mice in part C (p = 0.038). No other differences were significant. (B) Body mass measurements were obtained from 4–10 individual viable mice up to 6 days post-infection. Results were normalized to starting mass from individual mice and averaged; error bar represents SEM. (D-I) 9-week old male BALB/c mice were infected with 5x10^5^ of the indicated *C*. *albicans* strains. After 72 hrs post infection, kidney (D and G), liver (E, H) and spleen (F,I) were harvested and analyzed for CFUs as described in *Materials and Methods* (D-F) and Mn as in Fig 8 (G-I). Results are from 3 uninfected and 5–7 infected mice. Significance was determined by one-way ANOVA with a Tukey posttest. ****p<0.0001, **p<0.01, *p<0.05, ns p>0.05. Fungal strains utilized are as described in Fig 1D–1H.

Since host Mn decreases upon *C*. *albicans* invasion of the kidney, fungal acquisition of the metal may be critical for pathogen survival and virulence. To address this, we examined the effects of *C*. *albicans smf12Δ/Δ* and *smf13Δ/Δ* mutations on virulence in the mouse model for disseminated *candidiasis*. Mouse survival and weight loss was monitored in mice infected with wildtype versus the *smf* single mutants and the corresponding complemented strains. While wildtype *C*. *albicans* caused a lethal infection with an average survival time of 8 days (Fig 9A), mice infected with the *smf12Δ/Δ* and *smf13Δ/Δ* mutants had a statistically significant increase in median survival time of 16 and 21.5 days, respectively (Fig 9A). This loss in fungal virulence was fully rescued in the genetically complemented *smf12Δ/smf12Δ*:*SMF12* and *smf13Δ/smf13Δ*:*SMF13* strains where average mouse survival was not statistically different from wildtype *C*. *albicans* infected mice (Fig 9A). We additionally monitored body mass over the course of infection up to 6 days. As seen in Fig 9B, mice infected with wildtype *C*. *albicans* and the complemented *smf12Δ/smf12Δ*:*SMF12* and *smf13Δ/smf13Δ*:*SMF13* strains exhibited a more rapid decline in body mass compared to mice infected with *smf12Δ/Δ* and *smf13Δ/Δ* mutant strains, in line with mouse survival data. Consistent with the attenuated virulence, we observed that the number of colony forming units (CFUs) in the kidney were reduced by approximately 100-fold in the *smf12Δ/Δ* and *smf13Δ/Δ* infected mice (Fig 9D) and these mice exhibited a less pronounced host response in terms of kidney Mn loss (Fig 9G), indicating a link between severity of infection and the kidney response involving Mn. Compared to kidney, secondary sites of infection (liver and spleen) with 2–3 orders of magnitude lower CFUs showed no statistically significant differences in CFUs with WT *C*. *albicans* compared to the *smf* mutants (Fig 9E and 9F). There was also no statistically significant difference in liver or spleen Mn upon infection with WT versus *smf12Δ/Δ* and *smf13Δ/Δ* mutants (Fig 9H and 9I), although liver did exhibit a trend towards lower Mn with infection by all three fungal strains (Fig 9H) in line with previous studies [70]. In addition to these infection studies involving the single *smf12Δ/Δ* and *smf13Δ/Δ* mutants, we compared the virulence of the double *smf12Δ/Δ smf13Δ/Δ* mutant versus the single *smf12Δ/Δ* and *smf13Δ/Δ* strains. As seen in Fig 9C, the double *smf12Δ/Δ smf13Δ/Δ* shows a further decrease in virulence, particularly in comparison to the *smf12Δ/Δ* mutant. This additive effect of *smf12Δ/Δ* and *smf13Δ/Δ* mutations during *in vivo* infection corroborates the notion that Smf12 and Smf13 work independently in uptake of Mn nutrients for fungal fitness and virulence.

## Discussion

The findings here document the critical role Mn plays in fungal pathogenesis and in the host response to fungal invasion. We describe the identification of *C*. *albicans* Smf12 and Smf13 as two NRAMP transporters that function in the acquisition of Mn needed to activate Mn-requiring metalloenzymes such as Mn-SODs and MNT enzymes in the Golgi. The Mn derived from Smf12 and Smf13 is important for maturation of proteins in the secretory pathway (including the extracellular Cu-only SOD5), for assembly of the mannoprotein cell wall layer, for morphogenesis and for virulence. Our studies also describe a new host response to infection involving a decrease in tissue Mn during fungal invasion of the kidney. In this host environment, the Mn transporters Smf12 and Smf13 promote fungal survival.

It is intriguing that *C*. *albicans* employs two NRAMP transporters for physiological Mn accumulation. This situation is not unique, in that bakers’ yeast *S*. *cerevisiae* also has dual NRAMP transporters for Mn [29–32]. In the case of *C*. *albicans*, the two transporters partially compensate for one another under physiological conditions, since the single mutants are universally less severe than the double mutants with regard to defects in Mn accumulation (Fig 1D), Mn-dependent enzyme activity (Figs 2B and 3A) and hyphal morphogenesis (Fig 4B). Under high Mn conditions, Smf13 can fully compensate for loss of Smf12, since Mn accumulation in the *smf12Δ/Δ* single, but not *smf12Δ/Δ smf13Δ/Δ* double mutant is restored to WT levels with 10 μM extracellular Mn (S3 Fig). However, the converse is not true and the presence of *SMF12* does not restore WT levels of Mn accumulation to *smf13* mutants under the same high environmental Mn (S3 Fig). We conclude that Smf13 functions under a higher range of extracellular Mn than Smf12, helping to explain the rationale for dual transporters. We also suggest that the two transporters may play differential roles under different environmental conditions and indeed we observed that under low phosphate, Smf12 appears more critical for Mn uptake than Smf13 (Fig 3E). The animal host is a complex environment with numerous micro niches varying in bioavailability of nutrients including metals. Having dual NRAMP transporters that operate under disparate conditions provides a backup system for accumulation of this essential micronutrient.

We document for the first time a host response to infection involving whole tissue lowering of Mn. Kidney Mn drops 25–40% during infection with WT *C*. *albicans* (Fig 8B). By comparison, this response was attenuated or eliminated with the less virulent *C*. *albicans smf12Δ/Δ* and *smf13Δ/Δ* strains (Fig 9G), potentially due to the lower fungal burden with these mutants (Fig 9D). How might kidney Mn be lowered during infection with WT *C*. *albicans*? Mn levels in kidney can be controlled by three transporters, ZIP8 (*SLC39A8*), ZIP14 (*SLC39A14*) and ZNT0 (*SLC30A10*) [71]. Of these, mutations in ZIP8 lead to decreases in kidney Mn [72], and have been linked to increased susceptibility to pneumococcal infection and chemically induced inflammatory bowel disease [73, 74]. In addition to these transporters that affect whole tissue Mn, macrophages specifically express a Mn and Fe transporting NRAMP1 in the phagolysosome believed to deplete this compartment of essential metals and help guard against infection from intracellular pathogens such as *Mycobacteria*, *Leishmania* and *Salmonella* [75]. However, no such role has been established for NRAMP1 in disseminated *candidiasis*. Our infection model involves BALB/c mice with a defective NRAMP1 allele, and yet host Mn still appears limiting for the fungal pathogen based on the poor virulence of *smf12Δ/Δ* and *smf13Δ/Δ* mutants. Aside from these various host metal transporters, the metal binding immune protein calprotectin is released in the kidney at sites of *C*. *albicans* invasion and is predicted to limit the local availability of Mn for the pathogen [37, 76].

The Mn-deficient *smf12Δ/Δ* and *smf13Δ/Δ* mutants exhibit a clear loss in virulence, likely due to a number of independent defects. First, the defects in hyphal morphology associated with these mutants may impair tissue invasion; indeed other *C*. *albicans* mutants with morphological deficits show loss in virulence in the disseminated *candidiasis* mouse model [77]. Inhibition of Mn-MNT activity may impair function or localization of mannosylated proteins needed for virulence such as Cu-only SODs [57, 61]. The thick mannose layer of the cell wall plays an important role in immune recognition [78, 79], and the defects in cell surface mannosylation could conceivably impact the immune response [78, 79]. Defects in mannosylation might also directly affect fungal survival by inducing a protein unfolded stress response (UPR), as has been shown for chemical blockage of glycosylation with tunicamycin and rhodanine-3-acetic acid derivatives [80]. However, as seen in S5 Fig, the *smf12Δ/Δ* and *smf13Δ/Δ* mutants show no evidence for UPR as indicated by lack of up-regulation of the hallmark UPR stress markers *SEC61*, *KAR2* and *YSY6* [81, 82]. Aside from mannosylation, the loss in Mn-SOD activity may contribute to the virulence defect, as well as interruptions in any number of the many enzymes and pathways that rely on Mn. Regardless of the precise molecular defect(s), it is clear that *C*. *albicans* relies on Mn for virulence. These studies are the first to demonstrate a role for Mn at the host-fungal pathogen nexus and it should be interesting to test whether our findings can be extrapolated to numerous other pathogenic fungi and infection paradigms.

## Materials and methods

### Ethics statement

Animal studies were carried out under the National Institutes of Health guidelines for the ethical research involving animals. Experiments with mice were approved by the Institutional Animal Care and Use Committee (IACUC) of Johns Hopkins University (protocol number MO19M230), following guidelines of the Animal Welfare Act, The Institute of Laboratory Animal Resources Guide for the Care and Use of Laboratory Animals, and the Public Health Service Policy.

### Strains, Plasmids and Growth conditions

The *C*. *albicans* strains utilized in this study are in the background of the clinical isolate SC5314 (*smf11Δ/Δ*, *smf12Δ/Δ* and *smf13Δ/Δ* mutants), or in strain backgrounds derived from SC5314, including DAY286 (*smf12Δ/Δ* and *mnn9Δ/Δ* mutants), SN250 (*och1Δ/Δ* mutant), or CA-IF100 (*sod2Δ/Δ* and *sod3Δ/Δ* mutants). For details see S1 Table. Strains in the DAY286 and SN250 backgrounds were obtained through the Fungal Genetics Stocks Center [83–85]. The *C*. *albicans* CA-IF100, *sod2Δ/Δ* and *sod3Δ/Δ* strains were kind gifts of Karl Kuchler [58].

Homozygous deletions in the *SMF11*, *SMF12* and *SMF13* genes were generated in SC5314 using a CRISPR protocol [48] essentially as we have previously done [35].This procedure involves a split marker strategy to integrate Cas9, a nourseothricin (NTC) resistance marker, and a CRISPR guide template into the *HIS1* locus using two fragments of DNA, along with a 100 bp donor DNA oligonucleotide comprised of 50 bp upstream and 50 bp downstream of the coding region for *SMF11*, *SMF12* or *SMF13*. Benching Software was used to design guide RNAs. All oligonucleotides are listed in S2 Table. Positive transformants were selected on YPD media (see ahead) containing 200 or 400 μg/ml NTC, and gene deletions were confirmed by PCR and DNA sequencing, creating strains AW001 (*smf11Δ/Δ*), AW002 (*smf12Δ/Δ*) and AW003 (*smf13Δ/Δ*). In the case of AW002 and AW003, NTC sensitive derivatives were obtained by growth in 5 mls of media containing maltose (YPM see below) to stationary phase and by selecting NTC sensitive colonies that exhibited slow growth on 25 μg/ml NTC. The NTC sensitive *smf12Δ/Δ* strain was used to generate the *smf12Δ/Δ smf13Δ/Δ* double mutant (AW004) through a second round of CRISPR with *SMF13*. NTC sensitive *smf12Δ/Δ* and *smf13Δ/Δ* derivatives were used to create *SMF12+* and *SMF13+* rescue strains through genetic complementation as described below.

While engineering recombinant *SMF12* and *SMF13* for genetic complementation, we observed toxicity to *E*. *coli* with plasmid born full length *SMF*. Therefore, we designed a strategy that uses *SMF12* and *SMF13* generated by PCR and a split marker strategy similar to that used for CRISPR. All oligonucleotides are listed in S3 Table. In this case, transformation involves two DNA fragments that contain either the N- or C-terminal half of *SAT1* and *SMF* sequences for directed insertion at the corresponding *SMF12* or *SMF13* locus (summarized in S6 Fig). The fragment containing *SAT1* C-terminus and full length *SMF12* or *SMF13* was generated by PCR stitching of two smaller *SMF* containing segments obtained as follows: The upstream segment (denoted SMF-up in S6 Fig) spans *SMF12* residues -156 to +1215 or *SMF13–*156 to +1202 generated by PCR amplification of genomic DNA. The downstream segment (denoted SMF-down in S6 Fig) was derived by first inserting *SMF12* +800 to +2025 or *SMF13* +941 to +2157 into the SacI and NotI sites of pSF2 [35], yielding plasmids pAW001 (SMF12) or pAW002 (SMF13) (S6 Fig). Next, these plasmids were used as template to amplify the SMF-down segment spanning the C-terminal half of *SMF12* (from +1185) or *SMF13* (from +1180) to the C-terminus (+432) of *SAT1* (S6 Fig). Stitching of SMF-up and SMF-down together by PCR using either Pfusion (ThermoFisher Scientific) or Q5 (New England Biolabs) polymerase, yielded a fragment containing full length *SMF12* or *SMF13* and the C-terminal half of *SAT1* (S6 Fig). To generate the second fragment containing the N-terminal half of *SAT1* and *SMF* downstream sequences for targeted insertion, *SMF12* sequences +2013 to +2214 or *SMF13* sequences +2100 to +2263 (note: *SMF13* and *SMF13* stop codons are at +1755 and +1956) were inserted into Xho1 and Apa1 sites of pSF2 generating pAW003 and pAW004. The plasmids were then digested with BanII, creating a fragment that spanned *SMF12* +2214 or *SMF13* +2263 to *SAT1* residue +832 (S6 Fig). The two fragments containing the N- and C-terminal halves of *SAT1* were used to transform the corresponding *smf12Δ/Δ* or *smf13Δ/Δ* mutants and NTC resistant colonies selected. Proper insertion of a single copy of *SMF12* and *SMF13* was confirmed by PCR, producing the reintegrated rescue strains *smf12Δ/smf12Δ*:*SMF12* (AW005) and *smf13Δ/smf13Δ*:*SMF13* (AW006), respectively. NTC sensitivity of these rescue strains was restored by growth on maltose as described above.

All strains were typically maintained on YPD, a yeast extract, peptone-based media (1% yeast extract, 2% peptone, 2% dextrose). A low phosphate media was prepared using 0.56% yeast nitrogen base minus phosphate (MP Bio) supplemented with 2% glucose. To induce morphogenesis to the hyphal state, 3 ODs of cells grown in YPD to OD_600_ 2–4 were first starved in 1 mL of milli-Q water at 30°C for 30 min, then seeded in either Iscove Modified Dulbecco Media (IMDM, Thermofisher Scientific) or fetal bovine serum (Corning, heat inactivated) at 0.2 OD_600_ cell units/ mL. Hyphal cells developed by growth at 37°C, 220 RPM for various time points. YPM media (1% yeast extract, 2% peptone, 2% maltose) was used to stimulate FLP recombinase activity and to recover NTC resistance as described [48]. Toxicity tests with paraquat (methyl viologen, Sigma-Aldrich) were conducted in 1.0 ml YPD cultures seeded at an OD_600_ 0.0001 and grown for 20 hrs (14–18 cell doublings). For CFU determinations, infected tissues were weighed, homogenized and serially diluted prior to plating on YPD with 1% (volume:volume) Penicillin Streptomycin (Thermo Fisher Scientific).

### Biochemical analyses and microscopy

Analysis of metals was carried out on cell and tissue samples digested in nitric acid. Approximately 20 OD_600_ units of yeast-form cells or hyphal cells collected from 30 mL IMDM or 10 mL serum cultures were harvested by centrifugation at 4000 x g for 5–15 min (hyphal cells required longer times). Cells were washed in 1 mL of TE buffer (10 mM Tris, 1 mM EDTA, pH 8.0) and twice with 1 mL of milli-Q water. Prior to the final wash, cell number was determined at OD_600_. The cell pellet was resuspended in 100 μL of 10% optima grade nitric acid (Fisher Chemical). Whole kidneys, liver or spleen tissues were weighed and washed with TE and milli-Q water, then digested in 500 μL 20% nitric acid. Nitric acid digestions of fungal cells or tissues were performed at 90°C overnight. Samples were centrifuged to remove debris, and diluted to a final concentration of 2% nitric acid before metal analysis. Mn and Cu content were measured using an AAnalyst graphite furnace atomic absorption spectrometer (AAS) (PerkinElmer) and Zn was determined by inductively coupled plasma-mass spectrometry (ICP-MS, PerkinElmer NexION 300D, UMBC MCAC). Fe content in these samples was measured using a bathophenanthroline disulfonate (BPS) absorbance based assay as previously described [35].

Analysis of mRNA through qRT-PCR analysis involved 7 OD_600_ cell units of yeast-form *C*. *albicans* grown to an OD_600_ of 2–3 in YPD. RNA was extracted through the acid-phenol method as previously described [40]. DNase treatment involved the use of RapidOut DNA Removal kit (ThermoScientific), and cDNA synthesized using a Revert Aid First Strand cDNA synthesis kit (ThermoScientific)) prior to qRT-PCR with PowerUp SYBR Green Master Mix (ThermoScientific). Relative expression by normalizing to *TUB2* was calculated through the Δ*C*_*T*_ method as previously described [40]. The following primers were used to obtain Amplicons of ∼200 bp: *TUB2*, ATACGTTCCTCGTGCCGTTT and AACATTGCCGGCAGAACTTTG; *KAR2*, CTGAAGATTACCTTGGCAAAAAT and TTAGTA GCTTGTCTTTGAGCATCGTT; *YSY6*, ACACCTAAACAAAGAGCAGCTAATG and TTGCTCCACCACATACTAAGAA; *SEC61*, GTCACAGAGACACTTCTGCTTACAA and TAGACGTACCAGAACCAAGAGTACC.

Preparation of whole cell lysates for analysis of intracellular fungal Sods was carried out essentially as described [40]. Briefly, 20 OD_600_ cell units of yeast-form cells or 15 mLs of hyphal cells from IMDM cultures were harvested by centrifugation at 4000 x g. Cells were washed with 1 ml milli-Q water and pellets resuspended in 150 μl of a lysis buffer containing 5 mM EDTA, 5 mM EGTA, 50 mM NaCl, 10% glycerol, and 0.1% Triton X-100 in 10 mM sodium phosphate, pH 7.8. An equal volume of 0.5 mm zirconium oxide (ZO) beads (Research Products International) was added and the cells were lysed by BeadBlaster (Benchmark) at the maximum speed at 4°C for three one minute intervals between 30 second cooling periods. Samples were then centrifuged for 10 minutes at 14,000 x g. Supernatant containing soluble protein was retained and protein concentration was determined using the BioRad Protein Assay Dye.

Samples to survey the glycosylation state of extracellular Sod5 were obtained from hyphal cells as described [53]. A 15 mL IMDM culture grown at 37°C for 16 hrs was subjected to centrifugation at 4000 x g for 15 minutes. The cell pellet was resuspended in milli-Q water and transferred into a pre-weighed eppendorf tube for centrifugation at 14,000 x g for 5 min. Cells were washed with 1 mL of lyticase buffer (50 mM Tris pH 7.4, 2 mM PMSF with Pierce protease inhibitor (Thermo Scientific) and dried pellets weighed. Cell pellets of approximately 200 mg were resuspended in 150 μL lyticase buffer and subjected to ZO bead homogenization as above. The post-lysis material was subjected to centrifugation at 14,000 x g for 10 min, and the pellet containing cell wall material was washed once in the same lysis buffer and resuspended in 150 μL of lyticase buffer containing 30 units/mL *Arthrobacter luteus* lyticase (Millipore Sigma) to release GPI-anchored proteins from cell wall beta glucans. Following incubation at 30°C for 3 hrs, the reaction was centrifuged at 14,000 x g for 10 min and the supernatant was retained as the fraction containing cell wall Sod5. Where indicated, cell wall Sod5 containing samples were subjected to deglycosylation by PNGase F. 60 μL of wall material was digested with PNGase F (New England Biolabs) in a 100 μL reaction per manufacturer instructions.

Immunoblot analyses of intracellular SODs and extracellular Sod5 essentially followed published procedures [53, 86]. Gel electrophoresis was carried with 10 μg and 30 μg of protein from yeast-form and hyphal cell lysates respectively for analysis of intracellular Sods, or with proteins released from 5 and 30 mg of PNGase-treated and untreated samples for cell wall Sod5. Less protein was used for PNGase treated samples as Sod5 antibody recognition improves following deglycosylation [62]. In all cases, denaturing gel electrophoresis was carried out on 4–12% Bis-Tris acrylamide gels (Novex) at 200V for 35 minutes. Following electrophoresis, proteins were transferred to a PVDF membrane using the iBlot dry transfer system and blocked in 5% non-fat milk (VWR Life Science). Blots were probed with either anti-Sod1 at 1:10,000 dilution, or anti-Sod2 or anti-Sod3 at 1:5000 dilution or anti-Sod5 antibody at 1:5,000 dilution [53, 86], followed by goat anti-rabbit IgG Alexa Flour 680 secondary antibody at 1:10,000 (Thermofisher Scientific). Blots were imaged on an Odyssey infrared imaging system (LI-COR Biosciences) at 700 nm.

For SOD activity analysis, 5 μg and 20 μg of cellular lysate protein from yeast-form or hyphal cells were subject to native and non-reducing electrophoresis on 10% Tris-glycine gels (Novex) at 50 mA for 90 minutes at 4°C. Gels were then stained with nitroblue tetrazolium for 1 hour in the dark followed by destaining in H_2_O and imaging as previously described [86].

Alcian Blue binding assays for quantitating cell surface phosphomannans were performed essentially as described [27]. 0.5 OD_600_ cell units of yeast-form cells from log phase YPD cultures were harvested and washed once with 0.02M HCl. Cells were then pelleted and incubated with 1 mL of 30 μg/mL Alcian Blue (Sigma Aldrich) in 0.02 M HCl for 10 min at room temperature. Cells were harvested at 4000 x g and the supernatant collected. The Alcian Blue that did not bind to the cell surface was measured in the supernatant by absorbance at 620 nm and used to calculate the amount Alcian Blue bound to cells.

Intracellular acid phosphatase activity was surveyed as described [27]. Cells were grown in low phosphate media to OD_600_ from 2–5; approximately 10 OD_600_ cell units were harvested, washed with milli-Q water and resuspended in 200 μL of a lysis buffer containing 62.5 mM Tris-HCl (pH 6.8), 1 mM EDTA, 0.1 mM dithiothreitol, and Pierce protease inhibitor (Thermo Scientific). Cells were then subject to ZO bead homogenization as described above. Samples were centrifuged for 10 minutes at 14,000 x g, and the supernatant containing soluble protein was retained and protein concentration determined via BioRad Protein Assay. 85 μg of protein was digested with Endo H (endoglycosidase H, New England Biolabs) for 16 hours at 37°C in Glycobuffer 3 per manufacturer’s instructions. 20 μg of untreated or Endo H treated lysate protein was subjected to electrophoresis onto a 6% Tris/glycine-polyacrylamide gel (Novex) under native nonreducing conditions for 6 hr at 125 V. The gel was washed for 10 min in 100 mM sodium acetate pH 5.2 at room temperature and then incubated in fresh sodium acetate buffer containing 0.05% α-naphthyl phosphate substrate for 30 min at 37°C, followed by staining at 60°C with 0.03% fast blue, 0.05% α-naphthyl phosphate, 100 mM sodium acetate pH 5.2 for 10–25 min or until the colorimetric product developed. Gels were imaged and then stored in dH_2_O.

All microscopic analyses of fungal cells were carried out using dark field microscopy on a Nikon Infinity 1 microscope at 40X magnification. Germ tube length of hyphal cells was determined using ImageJ at a ratio of 1.5 μm per 10 pixels.

### Model of systemic candidiasis

Fungal cells to be used for infection were derived from yeast-form cultures of *C*. *albicans* grown to OD_600_ of 15.0 at 30°C, 220 rpm. To monitor Cu and Mn in host kidneys, 11 week -old female and male BALB/c mice were infected via a lateral tail vein injection with 5 × 10^5^ *C*. *albicans* SC5314 cells. Infected mice and non-infected controls were sacrificed at 72 hours and kidneys harvested for metal analysis. Tests for virulence involved 11 or 13 week -old male BALB/c mice infected via a lateral tail vein injection with 2 × 10^5^ *C*. *albicans* SC5314 and mutant strains *smf12Δ/Δ* (AW002), *smf13Δ/Δ* (AW003), *smf12Δ/Δ smf13Δ/Δ* (AM004), *smf12Δ/+* (AW005), and *smf13Δ/+* (AW006) cells. Seven to ten mice for each *C*. *albicans* strain were monitored daily for body mass and survival post-infection.

### Statistical analysis

Statistical Analyses were performed using GraphPad/ Prism9. The t-test was used for comparisons between two groups. And the one-way analysis of variance (ANOVA) along with a Tukey test was used for comparisons between groups of 3 or more. The Mantel Cox test was used for analysis of mouse survival post-infection for virulence studies. Quantification of SOD activity gels and immunoblots were carried out using ImageJ software.

## Supporting information

S1 Table *C*. *albicans* strains. (DOCX)

S2 Table Primers for CRISPR mutations and validation. (DOCX)

S3 Table Primers for creating *SMF12* and *SMF13* rescue. (DOCX)

S1 Fig Alignment of *C*. *albicans* Smf11, Smf12 and Smf13 versus *S*. *cerevisiae* Smf1. *S*. *cerevisiae* Smf1 was aligned against *C*. *albicans* Smf11, Smf12 and Smf13 using Clustal Omega software. Asterisks represent a fully conserved amino acid residue; one dots represent amino acid similarity and two dots represent amino acid identity and amino acid position are indicated in right margins.
(TIF)

S2 Fig Growth comparison of WT and *smf12Δ/Δ* and *smf13Δ/Δ* mutants. 1mL cultures of the indicated *C*. *albicans* strains were inoculated in YPD with a starting OD_600_ of 0.5 and grown in a 24-well plate for 16 hrs at 30°C with intermittent shaking in a BioTek Eon Microplate Spectrophotometer. Cell growth was monitored by plate reader absorbance at 600nm.
(TIF)

S3 Fig High extracellular Mn rescues the Mn deficiency of the *smf12Δ/Δ* and *smf13Δ/Δ* mutants. Total cellular Mn was measured in the indicated strains cultured in YPD (≈1 μM) or YPD supplemented with the indicated levels of MnCl_2_. Results from each graph are from three independent experimental trials. Significance was determined by one-way ANOVA with a Tukey posttest. ****p<0.0001, ***p<0.001, *p<0.05, ns p>0.05. Strains are as described in Fig 1D–1H.
(TIF)

S4 Fig Quantification of SOD protein levels and activity. Shown are quantification of Sod2 and Sod3 protein from immunoblots (A, B) and Sod1 enzymatic activity from native gels (C) of three-four independent experimental trials as described in Fig 2. (A) Analysis of samples from YPD grown cells cells as in Fig 2A and 2B; (B,C) Analysis of samples from IMDM grown cells as in Fig 2E and 2F. Results are normalized according to WT signals = 100%; strains are as described in Fig 1D–1H.
(TIF)

S5 Fig mRNA markers of the UPR response are not elevated in *smf12* and *smf13* mutants. mRNA levels of the unfolded protein stress markers *SEC61*, *KAR2* and *YSY6* in reference to *TUB2* were measured using q-RT PCR as described in *Materials and Methods*. Results are from four independent cultures over two experimental trials and are shown relative to that of the WT strain, the average of which is designated as 1.0. Strains are as described in Fig 1D–1H.
(TIF)

S6 Fig Strategy to engineer re-integrant strains. Cartoon depicting engineering of re-integrant strains as described in detail in *Materials and Methods*. In red are *SMF12* or *SMF13* sequences with position of START and STOP codons indicated. In blue are pSF2 plasmid sequences. SAT1; nourseothricin resistance marker; FLP, Flippase recombinase; F, FLP target sequence. Black arrows mark positions of primers used to amplify the fusion of downstream *SMF* sequences to C-terminal SAT1. DS, sequences downstream of *SMF* stop codon.
(TIF)

S1 Data Excel Sheets for Graphs. Excel spreadsheet containing in individual tabs, the raw numerical data used to generate the graphs in the following figure panels: Figs 1B, 1C, 1D, 1E, 1F, 1G, 1H, 2B, 2D, 2F, 3A, 3B, 3E, 4B, 4C,4D, 4E, 5A, 5B, 5C, 5D, 5E, 6A, 6B, 6C, 7B, 7D, 8A, 8B, 8C, 9A, 9B, 9C, 9D, 9E, 9F, 9G, 9H and 9I; S2, S3, S4 and S5 Figs.
(XLSX)
