# Supplementary material for: The role of manganese in morphogenesis and pathogenesis of the opportunistic fungal pathogen Candida albicans
Source: PLoS Pathog. 2023 Jun 26;19(6):e1011478. doi: 10.1371/journal.ppat.1011478 (PMC10328360; doi:10.1371/journal.ppat.1011478)
Supplement: S1 Table — (DOCX) [file ppat.1011478.s001.docx]

**S1 Table *C. albicans* strains**

| **Strain** | **Genotype** | **Source/ Ref** |
| --- | --- | --- |
| SC5314 | Clinical Isolate | Noble 2005 (1) |
| DAY286 | *ura3::limm434/ura3::limm434, ARG4:URA3::arg4::hisG /arg4∆::hisG, his1∆::hisG/: his1::hisG* | Nobile 2005 (2) |
| SN250 | *his1/his1, leu2::CdHIS1/leu2::CmLEU2, arg4/arg4, URA3/ura3:: limm434, IRO1/iro1:: limm434* | Noble 2010 (3) |
| CA-1F100 | *arg4/arg4, leu2/leu2::cmLEU2, his1/his1::cdHIS1, URA3/ura3∆* | Fronher 2009 (4) |
| CA-1F007 | *sod2::cmLEU2/sod2:: cdHIS1* in CA-1F100 | Fronher 2009 (4) |
| CA-1F011 | *sod3::cmLEU2/ sod3:: cdHIS1* in CA-1F100 | Fronher 2009 (4) |
| *mnn9∆* | *mnn9/mnn9* in DAY286 | Mitchell Collection FGSC |
| *smf12∆* | *smf12/smf12* in DAY286 | Mitchell Collection FGSC |
| *och1∆* | *och1::cmLEU2/och1::cdHIS1* in SN250 | Noble Collection GFSC |
| AW001 | *smf11∆/smf11∆, HIS1/his1::FRT* in SC5314 | this study |
| AW002 | *smf12∆/smf12∆, HIS1/his1::FRT* in SC5314 | this study |
| AW003 | *smf13∆/smf13∆, HIS1/his1::FRT* in SC5314 | this study |
| AW004 | *smf12∆/smf12∆, smf13∆/smf13∆, HIS1/his1::FRT* in SC5314 | this study |
| AW005 | *smf12∆/smf12∆::SMF12-FRT, HIS1/his1::FRT* in SC5314 | this study |
| AW006 | *smf13∆/smf13∆::SMF13-FRT, HIS1/his1::FRT* in SC5314 | this study |

1. Noble SM, Johnson AD. Strains and strategies for large-scale gene deletion studies of the diploid human fungal pathogen Candida albicans. Eukaryot Cell. 2005;4(2):298-309.

2. Nobile CJ, Mitchell AP. Regulation of cell-surface genes and biofilm formation by the C. albicans transcription factor Bcr1p. Curr Biol. 2005;15(12):1150-5.

3. Noble SM, French S, Kohn LA, Chen V, Johnson AD. Systematic screens of a Candida albicans homozygous deletion library decouple morphogenetic switching and pathogenicity. Nat Genet. 2010;42(7):590-8.

4. Frohner IE, Bourgeois C, Yatsyk K, Majer O, Kuchler K. Candida albicans cell surface superoxide dismutases degrade host-derived reactive oxygen species to escape innate immune surveillance. Mol Microbiol. 2009;71(1):240-52.
