# Supplementary material for: The role of manganese in morphogenesis and pathogenesis of the opportunistic fungal pathogen Candida albicans
Source: PLoS Pathog. 2023 Jun 26;19(6):e1011478. doi: 10.1371/journal.ppat.1011478 (PMC10328360; doi:10.1371/journal.ppat.1011478)
Supplement: S2 Table — (DOCX) [file ppat.1011478.s002.docx]

**S2 Table. Primers for CRISPR mutations and validation**

| **Name** | **Sequence** | **Use** |
| --- | --- | --- |
| CRISPR “A” fragment Fwd | GAC GGC ACG GCC ACG CGT TTA AAC CGC C | CRISPR A fragment |
| CRISPR “A” fragment Rev | CCC GCC AGG CGC TGG GGT TTA AAC ACC G | CRISPR A fragment |
| CRISPR “B” fragment Fwd | CAA ATT AAA AAT AGT TTA CGC AAG | CRISPR B fragment |
| CRISPR “C” Fragment Fwd | TAA AGC TGC CAC AAG AGG TAT TTC | CRISPR C fragment |
| CRISPR “C” Fragment Rev | AGG TGA TGC TGA AGC TAT TGA AG | CRISPR C fragment |
| SMF11 Guide | CGT AAA CTA TTT TTA ATT TGT GCA CCA GCA GTT ATA CCC GGT TTT AGA GCT AGA AAT AGC | To target degradation of SMF11 |
| SMF12 Guide | CGT AAA CTA TTT TTA ATT TGA TCC TGG TAA TTA TGC AAC AGT TTT AGA GCT AGA AAT AGC | To target degradation of SMF12 |
| SMF13 Guide | CGT AAA CTA TTT TTA ATT TGG ATA TGT CTG ATA GAG AAG GGT TTT AGA GCT AGA AAT AGC | To target degradation of SMF13 |
| SMF11 Donor Fwd | CTG TAA AAA AAA CAC TTC TAA TAA CCA AAA AAA CTA TAT CAA TAC AGA TCA AAG TTC AAG TCA TAG TAG TTG AAT ACT GGA CGT AAT TGA CAC CAT ATT T | Repair template for *smf11∆* |
| SMF11 Donor Rev | AAA TAT GGT GTC AAT TAC GTC CAG TAT TCA ACT ACT ATG ACT TGA ACT TTG ATC TGT ATT GAT ATA GTT TTT TTG GTT ATT AGA AGT GTT TTT TTT ACA G | Repair template for *smf11∆* |
| SMF12 Donor Fwd | ATA TTC CAA AAA AAC AAC AAG AAT CTT TCA CTT GTG TAA CAC CTA AAG AAG TAG TAT TTG CTT TAT TAT TTC TAA TAT ACA AGA ATA TAA CTT AGC CTC A | Repair template for *smf12∆* |
| SMF12 Donor Rev | TGA GGC TAA GTT ATA TTC TTG TAT ATT AGA AAT AAT AAA GCA AAT ACT ACT TCT TTA GGT GTT ACA CAA GTG AAA GAT TCT TGT TGT TTT TTT GGA ATA T | Repair template for *smf12∆* |
| SMF13 Donor Fwd | TAA CGA ATT TCA CAA ACT TCA AGA CCA TCG TGG CAA GTA TAA AAA AAT ACA ATT ACA AAA ACA CCC GTG TGT GGG GGA TAT ATT TTA TAG CAT TTG GAA T | Repair template for *smf13∆* |
| SMF13 Donor Rev | ATT CCA AAT GCT ATA AAA TAT ATC CCC CAC ACA CGG GTG TTT TTG TAA TTG TAT TTT TTT ATA CTT GCC ACG ATG GTC TTG AAG TTT GTG AAA TTC GTT A | Repair template for *smf13∆* |
| SMF11 KO Validation Fwd | CAA CAC ATC CCA TGG GAG AA | To validate deletion of SMF11 |
| SMF11 KO Validation Rev | CGA AGA GGT AGT AGT GGT CA | To validate deletion of SMF11 |
| SMF12 KO Validation Fwd | GTC ATT GAC GTT CTT GAG CC | To validate deletion of SMF12 |
| SMF12 KO Validation Rev | GGG ACT ACT ACA ACA ACA GC | To validate deletion of SMF12 |
| SMF13 KO Validation Fwd | GGT AAC ACA GCA ATA TCG TC | To validate deletion of SMF13 |
| SMF13 KO Validation Rev | GGT GGC ACA TCA AAT TTT GC | To validate deletion of SMF13 |
