# Supplementary material for: The role of manganese in morphogenesis and pathogenesis of the opportunistic fungal pathogen Candida albicans
Source: PLoS Pathog. 2023 Jun 26;19(6):e1011478. doi: 10.1371/journal.ppat.1011478 (PMC10328360; doi:10.1371/journal.ppat.1011478)
Supplement: S3 Table — (DOCX) [file ppat.1011478.s003.docx]

**S3 Table. Primers for creating *SMF12* and *SMF13* rescue**

| **Name** | **Sequence** | **Use** |
| --- | --- | --- |
| SMF12 C-terminus cloning Fwd | TCT CTC GAG CTC TCA TTG GGT CTA CAG TG | Insertion of SMF12 +800 to +2025 pSF2 |
| SMF12 C-terminus cloning Rev | TGT GTG GCG GCC GCC ATT TCA TTT CTT CGT GTG | Insertion of SMF12 +800 to +2025 pSF2 |
| SMF12 3’HA Cloning Fwd | TCT CTC GGG CCC TGA ACA AAG ATG GTA C | Insertion of SMF12 +2013 to +2214 into pSF2 |
| SMF12 3’HA Cloning Rev | TCT CTC CTC GAG ATG AAG TTG ATG AAA AC | Insertion of SMF12 +2013 to +2214 into pSF2 |
| SMF12 N-terminus Fwd | ACT GGT TTT CCA AGT CAC TTT TTG GTG | Amplification of SMF12 -156 to +1215 |
| SMF12 N-terminus Rev | CAA TAA ATC ATG AAT AGT GTA TAA ATC GGC | Amplification of SMF12 -156 to +1215 |
| SMF12 Fusion to NAT Fwd | GCC GAT TTA TAC ACT ATT CAT GAT TTA TTG | Amplification of SMF12 +1185 to +432 of SAT1 off cloned product |
| SMF12 Fusion to NAT Rev | CAA CAT TGC TTT TGG TGT TTG | Amplification of SMF12 +1185 to +432 of SAT1 off cloned product |
| SMF12 Stitching Fwd | CTG GTT TTC CAA GTC AC | Fusion of full SMF12 gene to partial NAT marker |
| SMF12 Stitching Rev | CAA CAT TGC TTT TGG TG | Fusion of full SMF12 gene to partial NAT marker |
| SMF13 C-terminus cloning Fwd | TCT CTC GAG CTC TCA TTG GGT CTA CAG TG | Insertion of SMF13 +941 to +2157 pSF2 |
| SMF13 C-terminus cloning Rev | TCT CTC GCG GCC GCG AAA CTT GGA ACA AGG TG | Insertion of SMF13 +941 to +2157 pSF2 |
| SMF13 3’HA Cloning Fwd | TCT CTC CTC GAG CCA TCT AAT ACT GCT TGT TC | Insertion of SMF13 +2100 to +2263 into pSF2 |
| SMF13 3’HA Cloning Rev | TCT CTC GGG CCC GCT CCA AAA TAC CGT AGT | Insertion of SMF13 +2100 to +2263 into pSF2 |
| SMF13 N-terminus Fwd | CGT CAT TAC ACT TAA GAG GGT AAC | Amplification of SMF13 -156 to +1202 |
| SMF13 N-terminus Rev | GCT AAA GTA CAA ACA ACC CCT G | Amplification of SMF13 -156 to +1202 |
| SMF13 Fusion to NAT Fwd | CAG GGG TTG TTT GTA CTT TAG C | Amplification of SMF13 +1180 to +432 of SAT1 off cloned product |
| SMF13 Fusion to NAT Rev | CAA CAT TGC TTT TGG TGT TTG | Amplification of SMF13 +1180 to +432 of SAT1 off cloned product |
| SMF13 Stitching Fwd | CGT CAT TAC ACT TAA GAG | Fusion of full SMF13 gene to partial NAT marker |
