# Supplementary figures and images for: The role of manganese in morphogenesis and pathogenesis of the opportunistic fungal pathogen Candida albicans
Source: PLoS Pathog. 2023 Jun 26;19(6):e1011478. doi: 10.1371/journal.ppat.1011478 (PMC10328360; doi:10.1371/journal.ppat.1011478)

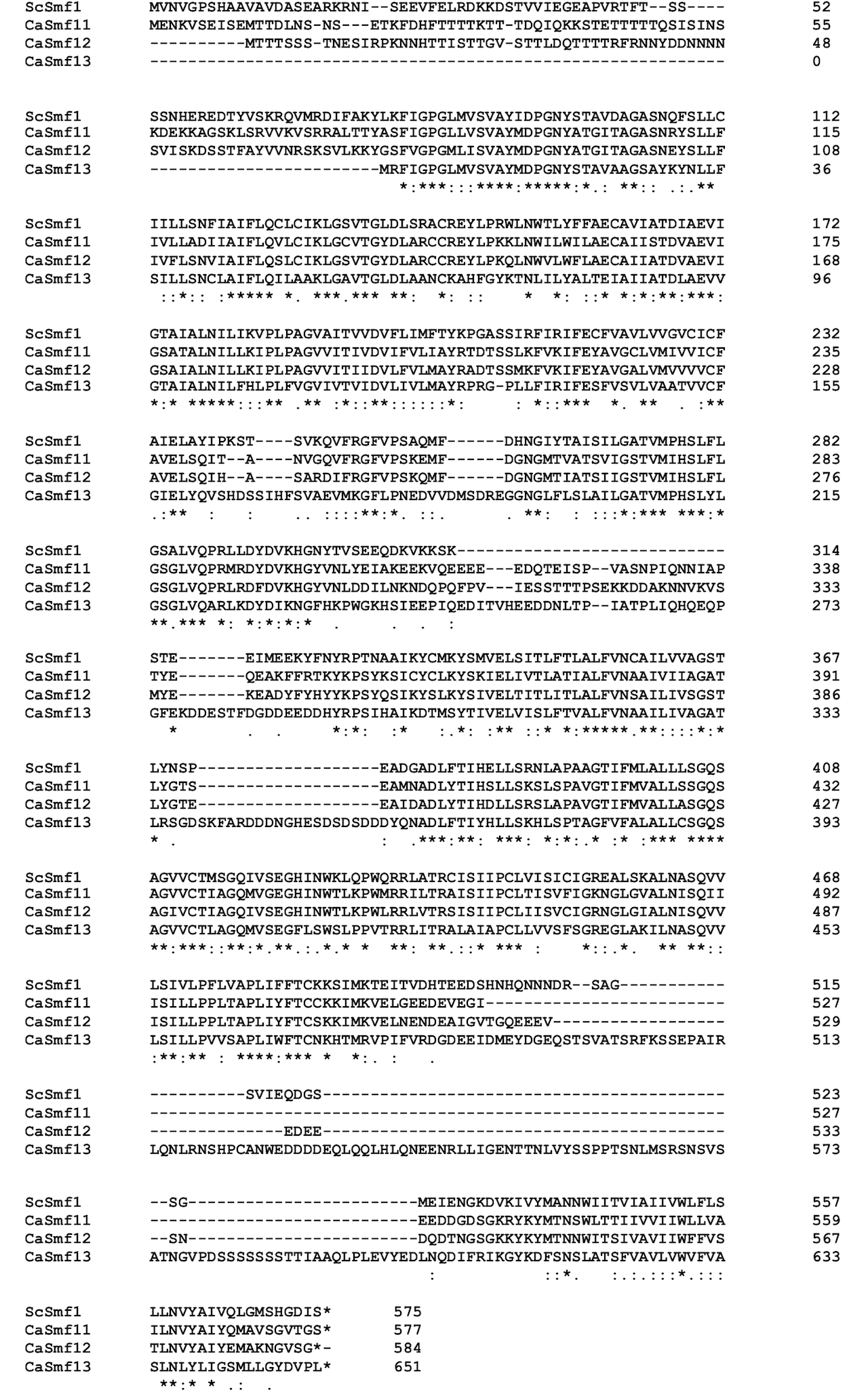

Supplement: S1 Fig — S. cerevisiae Smf1 was aligned against C. albicans Smf11, Smf12 and Smf13 using Clustal Omega software. Asterisks represent a fully conserved amino acid residue; one dots represent amino acid similarity and two dots represent amino acid identity and amino acid position are indicated in right margins. (TIF) [file ppat.1011478.s004.tif]

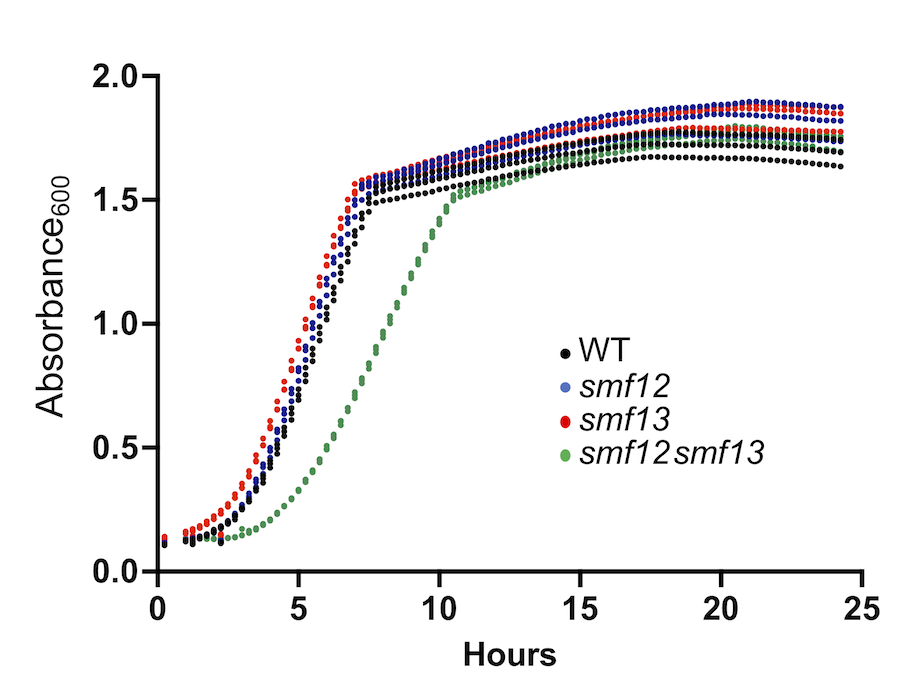

Supplement: S2 Fig — 1mL cultures of the indicated C. albicans strains were inoculated in YPD with a starting OD600 of 0.5 and grown in a 24-well plate for 16 hrs at 30°C with intermittent shaking in a BioTek Eon Microplate Spectrophotometer. Cell growth was monitored by plate reader absorbance at 600nm. (TIF) [file ppat.1011478.s005.tif]

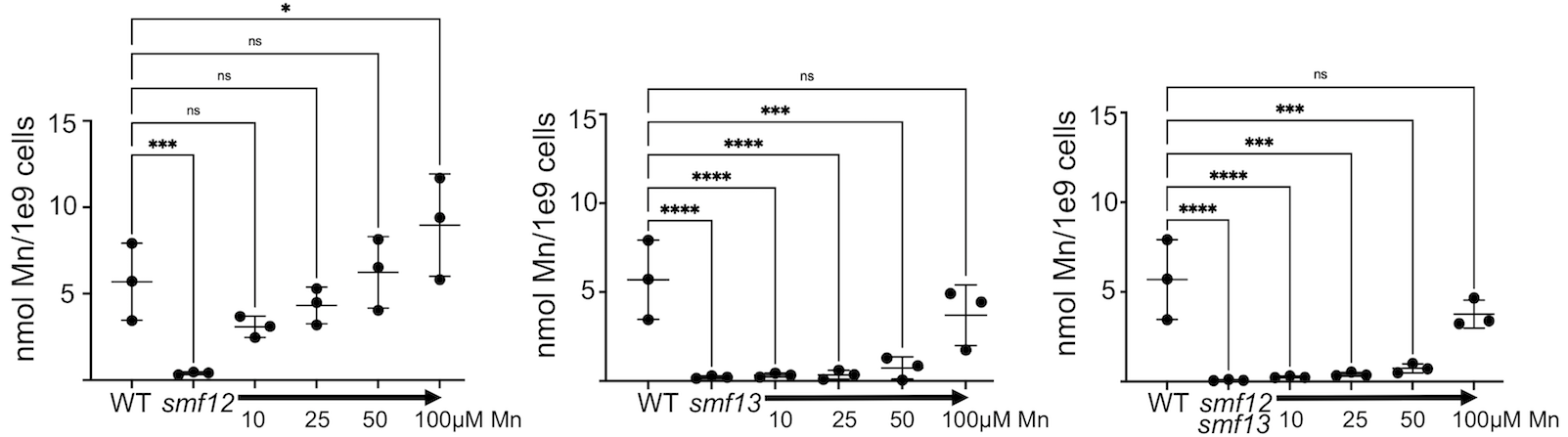

Supplement: S3 Fig — Total cellular Mn was measured in the indicated strains cultured in YPD (≈1 μM) or YPD supplemented with the indicated levels of MnCl2. Results from each graph are from three independent experimental trials. Significance was determined by one-way ANOVA with a Tukey posttest. ****p<0.0001, ***p<0.001, *p<0.05, ns p>0.05. Strains are as described in Fig 1D–1H. (TIF) [file ppat.1011478.s006.tif]

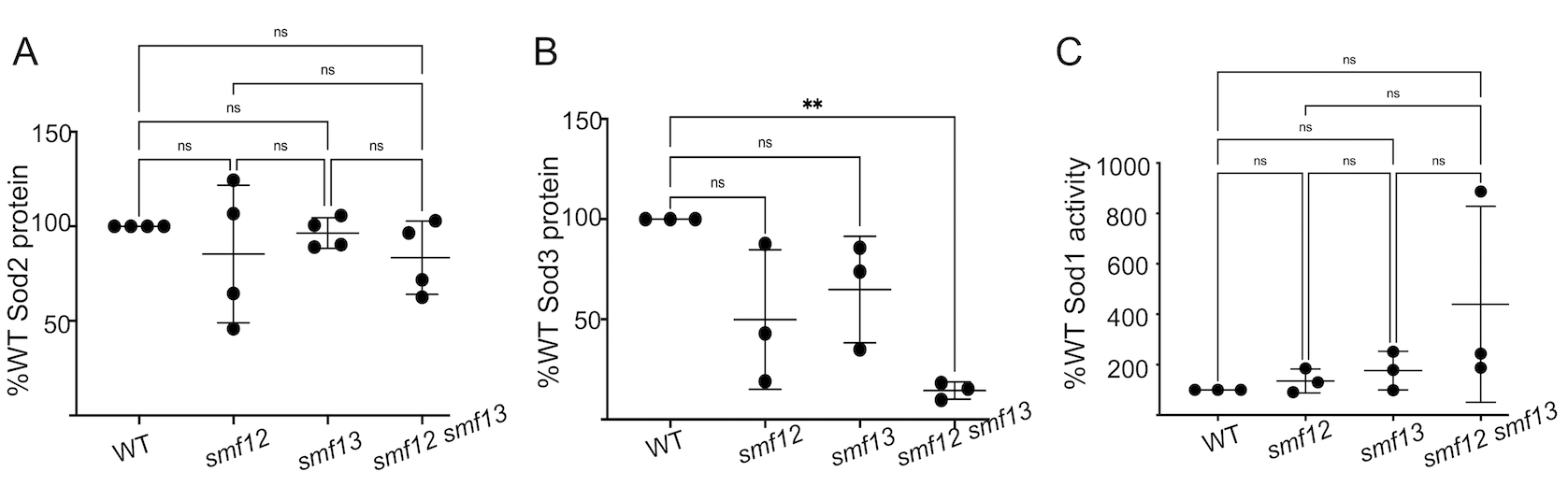

Supplement: S4 Fig — Shown are quantification of Sod2 and Sod3 protein from immunoblots (A, B) and Sod1 enzymatic activity from native gels (C) of three-four independent experimental trials as described in Fig 2. (A) Analysis of samples from YPD grown cells cells as in Fig 2A and 2B; (B,C) Analysis of samples from IMDM grown cells as in Fig 2E and 2F. Results are normalized according to WT signals = 100%; strains are as described in Fig 1D–1H. (TIF) [file ppat.1011478.s007.tif]

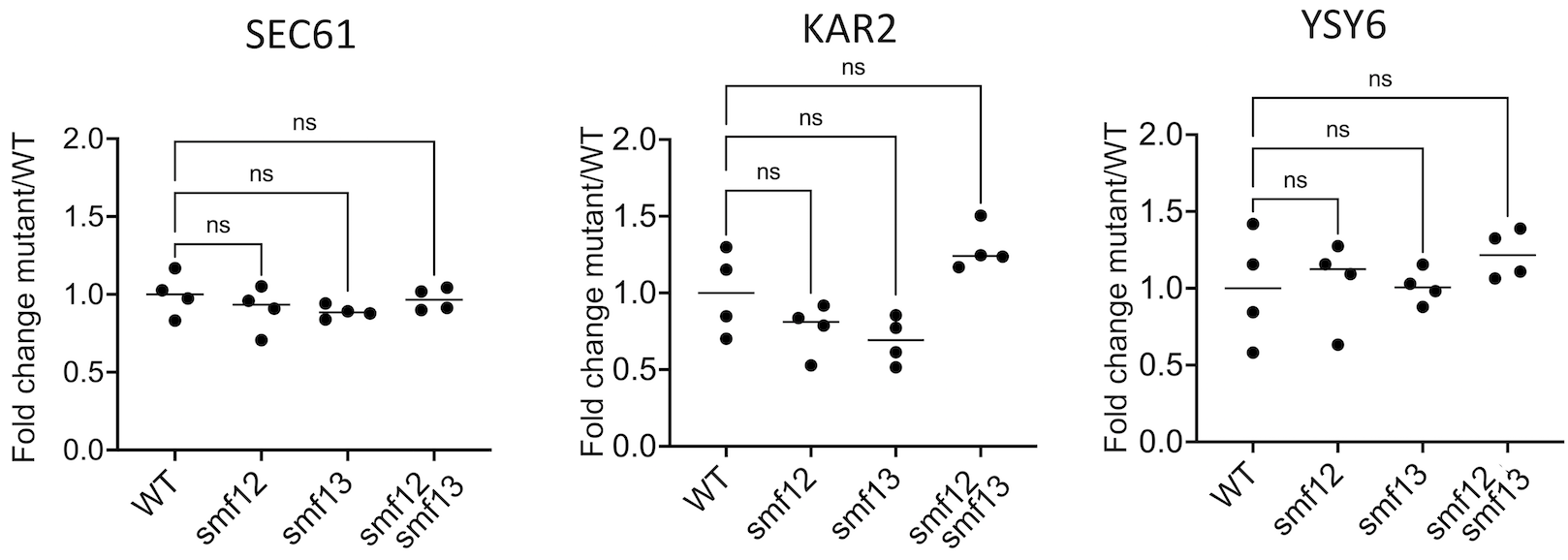

Supplement: S5 Fig — mRNA levels of the unfolded protein stress markers SEC61, KAR2 and YSY6 in reference to TUB2 were measured using q-RT PCR as described in Materials and Methods. Results are from four independent cultures over two experimental trials and are shown relative to that of the WT strain, the average of which is designated as 1.0. Strains are as described in Fig 1D–1H. (TIF) [file ppat.1011478.s008.tif]

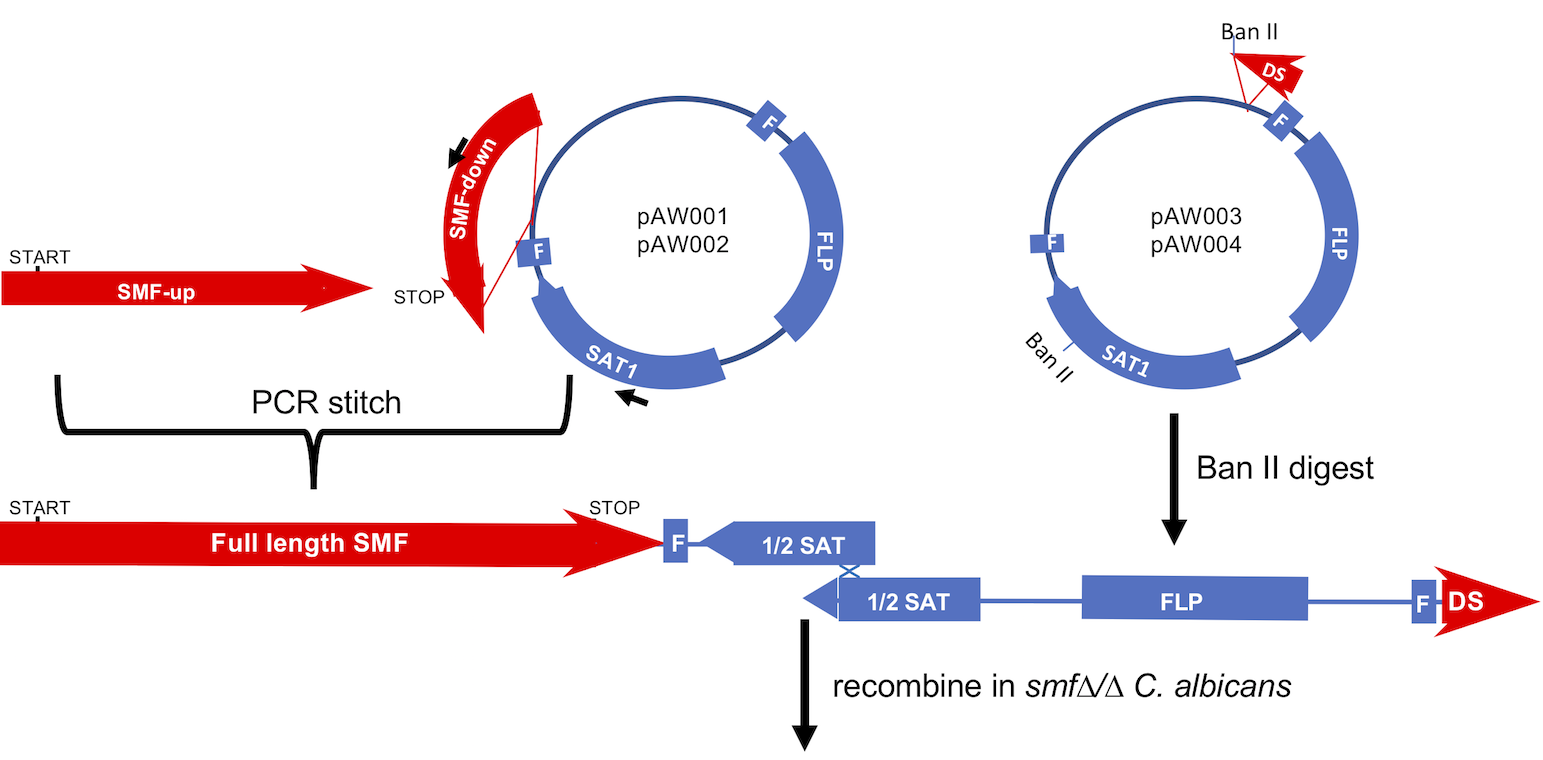

Supplement: S6 Fig — Cartoon depicting engineering of re-integrant strains as described in detail in Materials and Methods. In red are SMF12 or SMF13 sequences with position of START and STOP codons indicated. In blue are pSF2 plasmid sequences. SAT1; nourseothricin resistance marker; FLP, Flippase recombinase; F, FLP target sequence. Black arrows mark positions of primers used to amplify the fusion of downstream SMF sequences to C-terminal SAT1. DS, sequences downstream of SMF stop codon. (TIF) [file ppat.1011478.s009.tif]
